# Evidence for a prolonged Permian–Triassic extinction interval from global marine mercury records

**DOI:** 10.1038/s41467-019-09620-0

**Published:** 2019-04-05

**Authors:** Jun Shen, Jiubin Chen, Thomas J. Algeo, Shengliu Yuan, Qinglai Feng, Jianxin Yu, Lian Zhou, Brennan O’Connell, Noah J. Planavsky

**Affiliations:** 1 State Key Laboratory of Geological Processes and Mineral Resources, China University of Geosciences, 430074 Wuhan, Hubei China; 2 Department of Geology and Geophysics, Yale University, New Haven, CT 06520-8109 USA; 3 State Key Laboratory of Environmental Geochemistry, Institute of Geochemistry, Chinese Academy of Sciences, Guiyang, 550002 China; 4 Institute of Surface-Earth System Science, Tianjin University, 92 Weijin Road, 300072 Nankai, Tianjin China; 5 State Key Laboratory of Biogeology and Environmental Geology, China University of Geosciences, 430074 Wuhan, Hubei China; 6 Department of Geology, University of Cincinnati, Cincinnati, OH 45221-0013 USA

## Abstract

The latest Permian mass extinction, the most devastating biocrisis of the Phanerozoic, has been widely attributed to eruptions of the Siberian Traps Large Igneous Province, although evidence of a direct link has been scant to date. Here, we measure mercury (Hg), assumed to reflect shifts in volcanic activity, across the Permian-Triassic boundary in ten marine sections across the Northern Hemisphere. Hg concentration peaks close to the Permian-Triassic boundary suggest coupling of biotic extinction and increased volcanic activity. Additionally, Hg isotopic data for a subset of these sections provide evidence for largely atmospheric rather than terrestrial Hg sources, further linking Hg enrichment to increased volcanic activity. Hg peaks in shallow-water sections were nearly synchronous with the end-Permian extinction horizon, while those in deep-water sections occurred tens of thousands of years before the main extinction, possibly supporting a globally diachronous biotic turnover and protracted mass extinction event.

## Abstract (web summary)

Previously, little direct evidence has been found to link large volcanic eruption events with the end-Permian mass extinction. Here, the authors find that mercury enrichment and isotope records in marine sections across the globe can be linked to increased volcanic activity, which resulted in the protracted Permian-Triassic biocrisis

## Introduction

The mass extinction at the end of the Permian, ~252 million years ago, was the largest biocrisis of the Phanerozoic Eon and featured ~90% of marine invertebrate taxa going extinct in a geologically short time interval (~61 ± 48 kyr^1–3^). The main cause of the latest Permian mass extinction (LPME) is generally thought to be linked to severe environmental perturbations caused by eruptions of the Siberian Traps Large Igneous Province (LIP)^4,5^. Although the near-synchronous occurrence of increased volcanic activity and the LPME is well established^1,3,6^, geochemical evidence of a direct relationship between the LPME and the Siberian Traps LIP has been generated from only for a few marine sites in Arctic Canada and southern China (e.g., refs. ^7–11^).

Mercury enrichments in marine sedimentary successions represent a promising tool for identifying periods of enhanced volcanic activity. The modern global volcanic Hg flux is 76 ± 30 tons per year, accounting for 20–40% of total natural emissions of mercury^12^ (Supplementary Note 1). Combustion of coal can also result in a substantial Hg flux to the atmosphere, given its generally high Hg abundances (~500–1000 ppb^13^), and has been a major anthropogenic source of Hg throughout the industrial era^14^. However, volcanic activity (including direct gaseous emissions as well as magmatic intrusion into organic-rich sediments) was the major source of Hg to the atmosphere prior to the industrial era^12,14^. Massive volcanic eruptions (including LIPs) have frequently been associated with a spike in sedimentary Hg concentrations at geological boundaries, e.g., the Emeishan LIP (~260 Ma) at the Guadalupian–Lopingian Boundary^10^, the Siberian Traps (~252 Ma) at the Permian–Triassic boundary (PTB)^7,8,11^, the Central Atlantic Magmatic Province (CAMP, ~201 Ma) at the Triassic–Jurassic Boundary^15,16^, and the Deccan Traps LIP (~66 Ma) near the Cretaceous–Paleogene Boundary^17,18^.

**Fig. 1:**
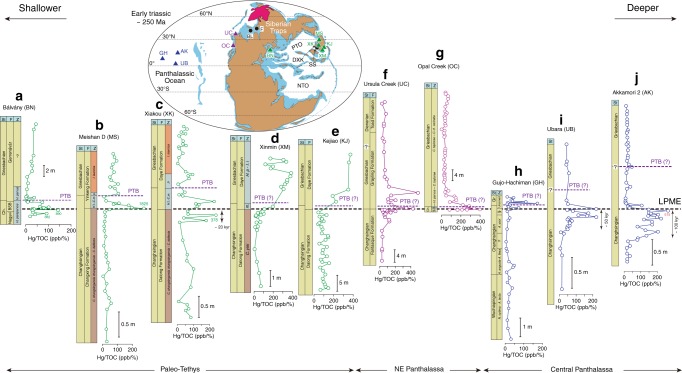
Mercury concentration profiles (Hg/TOC) of study sections. **a** Bálvány, **b** Meishan D, **c** Xiakou, **d** Xinmin, **e** Kejiao, **f** Ursula Creek, **g** Opal Creek, **h** Gujo-Hachiman, **i** Ubara, and **j** Akkamori 2. The red vertical double-arrow lines in Xiakou, Ubara, and Akkamori-2 sections represent time gaps between the initial Hg spike and the LPME. Section locations shown on Early Triassic (~250 Ma) global paleogeographic map (adapted from Ron Blakey, http://jan.ucc.nau.edu/~rcb7/). Triangles represent the sections analyzed in this study (green, purple, and blue represent sections from Paleo-Tethys, Neo-Tethys, and Panthalassic oceans, respectively), and black circles show previously investigated sections in northern Laurentia and the South China area (see text). *A*. *Albaillella*; *C.* *Clarkina*; *de*. *A. degradans*; *H.* *Hindeodus; H.l.-C.m*. *H. latidentatus-C. meishanensis*; *H.p-I.i* *=* *H. parvus-I. isarcica; I.* *=* *Isarcicella; m*. = *C. meishanensis*; *N.* *Neoalbaillella*; *p*. *H. parvus*; *sh.* *Mesogondolella sheni*; *tr*. *A. triangularis; yin.* *C. yini*. Ch. Changhsingian, Gr. Griesbachian; St (sub)stage; F formation; B bed; Z conodont zone (all sections except Gujo-Hachiman), and radiolarian zone (Gujo-Hachiman). Sections: AK Akkamori-2; BL Buchanan Lake; BN Bálvány; DXK Daxiakou; F Festningen; GH Gujo-Hachiman; KJ Kejiao; M Mud; MS Meishan; OC Opal Creek; SS Shangsi; UB Ubara; UC Ursula Creek; XM Xinmin; XK Xiakou. BSB boundary shale beds, PTO Paleo-Tethys Ocean, NTO Neo-Tethys Ocean. LPME latest Permian mass extinction, PTB Permian–Triassic boundary. Note: full geochemical data are in Supplementary Figures 2–11. Source data are provided as a Source Data file

Hg concentration spikes synchronous with the LPME have been reported from PTB sections at Festningen, Buchanan Lake, Shangsi, Daxiakou, and Meishan D^7,8,10,11^ (Fig. 1). These data suggest large inputs of volcanic Hg into the regions of northern Laurentia and the South China Craton^8,11^, though they are not sufficient to establish the timing of volcanic fluxes relative to the extinction horizon on a global scale. In this study, we measured Hg concentrations in 10 marine PTB sections and analyzed Hg isotopes in three of these sections (from different areas and depositional settings) to investigate if mass-dependent and mass-independent Hg isotope fractionations can provide insight into the origin and transport vectors of Hg during the Permian–Triassic transition.

## Results

### Study sections

The 10 PTB sections chosen for this study have a wide geographic distribution (Fig. 1) and represent a range of depositional water depths, including shallow (shelf or platform, <100 m), intermediate (deep shelf to upper slope, 100–1000 m), and deep water settings (abyssal, >2000 m) (see Shen et al.^19^; Supplementary Note 2 and Supplementary Fig. 1). These sections are assigned to four marine regions, including two deep-shelf sections (Opal Creek and Ursula Creek) in northeastern Panthalassa; one shelf section (Bálvány) in the western Paleo-Tethys; one shallow-shelf (Meishan) and three deep-shelf to slope sections (Xiakou, Xinmin, and Kejiao) in the eastern Paleo-Tethys (South China); and three abyssal sections (Gujo-Hachiman, Akkamori-2, and Ubara) in central Panthalassa (Fig. 1). These sections are correlated to each other based on conodont and radiolarian biostratigraphy, as well as carbon-isotope chemostratigraphy (see Methods). For most study sections, Hg analysis was constrained to the narrow PTB interval (ca. ±1 Myr, Supplementary Fig. 1), but some sections were analyzed over larger stratigraphic intervals, extending downsection to Wuchiapingian-age strata (Gujo-Hachiman) or upsection to Dienerian-age strata (Ursula Creek) (Supplementary Fig. 1). Three sections representing shallow (Meishan D), intermediate (Xiakou), and deep (Gujo-Hachiman) sites were chosen for Hg isotope analysis. For each study section, a detailed description and additional geochemical profiles are given in Supplementary Note 2 (Supplementary Figs. 2–11).

### Mercury concentrations

**Fig. 2:**
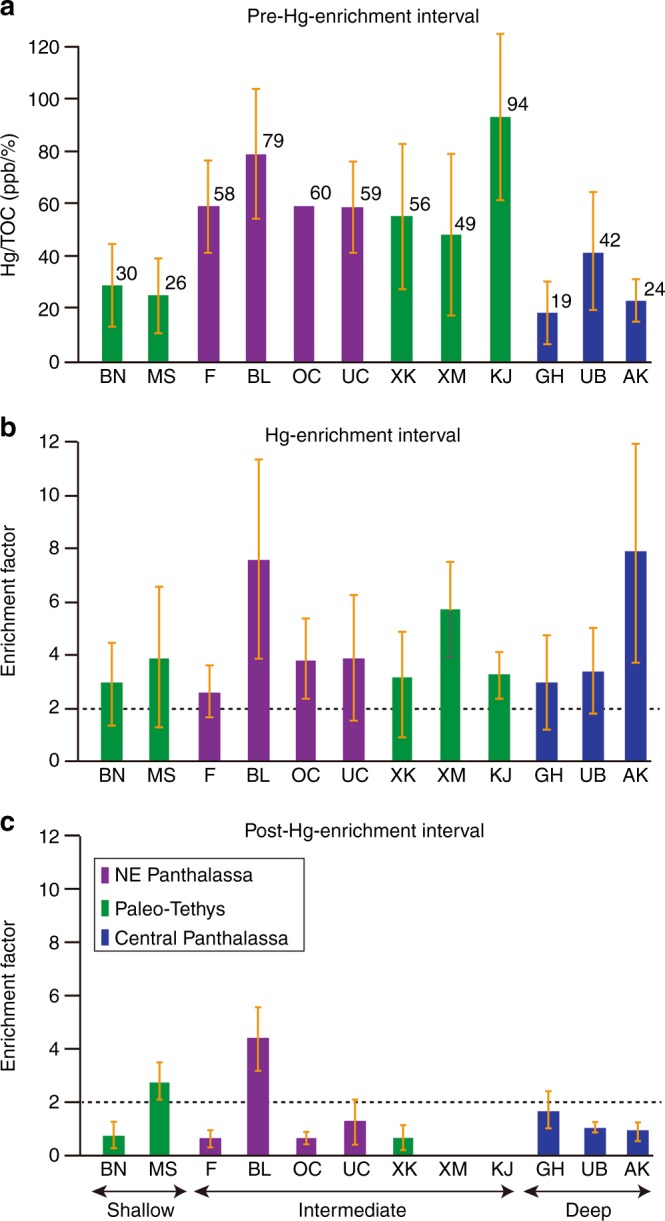
Mercury enrichment levels relative to latest Permian mass extinction. Average Hg/TOC ratios by study section for the pre-enrichment interval (**a**), and average enrichment factors (Hg-EFs) for the enrichment (**b**) and post-enrichment intervals (**c**). Vertical whiskers represent standard deviation (SD) ranges. The numbers in panel **a** represent the mean values of each section. Data for Buchanan Lake and Festningen are from refs. ^8,10^. The Opal Creek section lacked pre-LPME samples, so a pre-enrichment value of 60 ppb/% was assumed based on the mean values of the Festningen and Ursula Creek sections. See Fig. 1 for section abbreviations and Supplementary Figure 1 for depositional water depths. Refer to Supplementary Note 2 for detailed section descriptions. Source data are provided as a Source Data file

A total of 391 samples were analyzed for Hg concentrations, with 28–59 analyses per section (Figs. 1, 2). Because Hg is hosted mainly by organic matter^20^, Hg concentrations were normalized to total organic carbon (TOC) in order to discern enrichments independent of variations in TOC^21^. Background Hg/TOC ratios range from 20 at Gujo-Hachiman to 95 at Kejiao, with a mean of ~50 for the full dataset (Fig. 2a; note all Hg/TOC values have units of ppb/%). Relative to these baseline values, all 10 study sections exhibit a pronounced increase in Hg/TOC ratios close to the LPME and extending upsection some distance into the Lower Triassic (Figs. 1, 2). In order to assess secular variation in Hg enrichment, mean Hg/TOC values were calculated for pre-enrichment, enrichment, and post-enrichment intervals in each study section. The latter two values are expressed as enrichment factors (EF) relative to pre-enrichment Hg levels, where Hg-EF = (Hg/TOC)/(Hg/TOC)_bg,_ with ‘bg’ representing background (pre-enrichment) values. The enrichment interval extends from the sharp rise in Hg/TOC values close to the LPME upsection into the lowermost Triassic until the point that Hg/TOC falls to <1/2 of peak values. These operationally defined intervals correspond approximately to the pre-extinction, extinction, and post-extinction phases of the PTB crisis in shallow and intermediate sections (see below), where the extinction phase is delineated by the first and second extinction pulses at Meishan D^22,23^. We chose not to define enrichment intervals based on the extinction phases per se because the second extinction pulse (of lowermost Triassic age) has not been identified in many of the study sections.

**Fig. 3:**
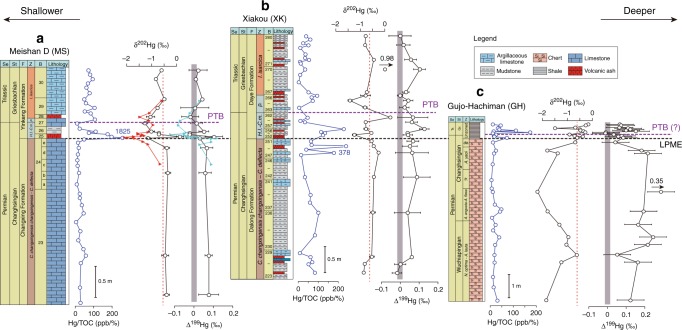
Mercury isotopes of selected study sections. Profiles ratios of mercury to total organic carbon (Hg/TOC), mass-dependent fractionation (δ^202^Hg), and mass-independent fractionation (Δ^199^Hg) for three sections: **a** Meishan D (triangles represent data from^8^); **b** Xiakou; and **c** Gujo-Hachiman. The red dashed lines and shaded rectangles are the background values of δ^202^Hg (‒0.65‰) and Δ^199^Hg (0‰), respectively. *A*. *Albaillella*; *C.* *Clarkina*; *de*. *A. degradans*; *H.l.-C.m*. *H. latidentatus-C. meishanensis*; *I.* *Isarcicella; N.* *Neoalbaillella; p*. *H. parvus*; *tr*. *A. triangularis*. Gr. Griesbachian, Tr. Triassic; Se Series, St (sub)stage, F formation, B bed, Z conodont zone (all sections except Gujo-Hachiman), and radiolarian zone (Gujo-Hachiman). LPME latest Permian mass extinction, PTB Permian–Triassic boundary. The horizontal bars of the isotope profiles indicate standard deviation (2*σ*) values, which are smaller than the symbol size for some samples. Source data are provided as a Source Data file

The pre-enrichment interval shows the lowest average Hg/TOC ratios, the enrichment interval the highest average ratios, and the post-enrichment interval intermediate and spatially more variable ratios (Fig. 2). For the enrichment interval, the largest EFs are observed at Akkamori-2 (8.2), Buchanan Lake (7.6), and Xinmin (5.7), while all 10 study sections (as well as two previously reported sections) exhibit EFs of >2 (Fig. 2b). For the post-enrichment interval, Buchanan Lake and Meishan D yield lower yet still significant EFs (4.3 and 2.8), but all of the remaining sections show declines in Hg-EF to <2 (Fig. 2c). Peak Hg/TOC levels are observed significantly below the LPME in the two deep-ocean sections at Akkamori-2 (‒0.5 m) and Ubara (‒0.3 m), implying the onset of Hg enrichment ~100 and ~50 kyr prior to the LPME, respectively, based on the age models for these sections^1,2^ (see Methods). The slope section at Xiakou exhibits an Hg/TOC peak preceding the LPME by ~20 kyr (Figs. 1, 3). In contrast, most shallow-water sections exhibit no gap between the Hg/TOC peak and the mass extinction horizon (e.g., both are in Bed 25 of the Meishan section). The one exception to this deep-vs.-shallow pattern is the deepwater Gujo-Hachiman section, where the initial Hg enrichment coincided with the mass extinction horizon, although we cannot rule out the possibility of a genuine signal, e.g., related to stratigraphic condensation or a hiatus around the LPME or, more simply, because of low sampling resolution in this section.

### Mercury isotopes

Hg isotopes were measured for sample subsets from Meishan D (*n* = 15), Xiakou (*n* = 21), and Gujo-Hachiman (*n* = 24). The Hg isotope profiles (δ^202^Hg tracking mass-dependent fractionation, and Δ^199^Hg tracking mass-independent fractionation) show pronounced secular changes around the LPME and PTB horizons (Fig. 3). At Meishan D, Beds 25 and 28 exhibit negative excursions in both δ^202^Hg (to ‒1.3‰ from background values of ‒0.4 to ‒0.6‰; Fig. 3a) and Δ^199^Hg (to ‒0.02‰ from background values of 0 to +0.08‰; Fig. 3a). Grasby et al. ^8^ reported similar secular trends at Meishan over a more limited stratigraphic interval (1.0 m vs. 3.5 m in the present study) (Fig. 3a). Although the numbers of samples at Xiakou (also called Daxiakou) are limited, two negative δ^202^Hg excursions are present in the *C. meishanensis* and *I. isarcica* Zones^22^ (to <‒1.4‰ from background values of ca. ‒0.6‰; Fig. 3b). Δ^199^Hg shows more limited deviations (to ca. +0.1‰ from background values of 0 to +0.07‰; Fig. 3b). These δ^202^Hg and Δ^199^Hg profiles are similar to profiles for Xiakou in an earlier study^11^. At Gujo-Hachiman, δ^202^Hg shows fluctuating but low pre-extinction values (ca. ‒2.1 to ‒1.0‰) followed by a sharp rise above the LPME (to ‒0.8 to 0‰; Fig. 3c). The Δ^199^Hg profile shows nearly the opposite pattern, with fluctuating but high pre-extinction values (+0.12 to +0.35‰) decreasing to ‒0.02 to +0.14‰ above the LPME (Fig. 3c). It should be noted that, owing to low sedimentation rates at Gujo-Hachiman^24^, the interval >40 cm below the LPME is stratigraphically older than the studied intervals at Meishan and Xiakou. The lower δ^202^Hg and higher positive Δ^199^Hg values of lower to middle Changhsingian strata at Gujo-Hachiman thus represent stable deep-marine sedimentation prior to the end-Permian crisis.

## Discussion

The accumulation of Hg in sediments can be influenced by various factors including redox conditions, organic carbon burial fluxes, and clay mineral content^16,20,25–27^. Reducing conditions promote the formation of organic-Hg complexes and Hg-sulfides, which are likely to be the dominant forms of Hg in marine sediments^20,26,27^. Hg can become enriched in clay minerals under certain chemical conditions (e.g., elevated E_h_) through adsorption of sparingly soluble Hg(OH)_2_^28,29^. However, Hg adsorbed onto organic matter is the dominant form of Hg in most aquatic systems^20^. Small mass-dependent fractionations (MDF) may result from physical–chemical–biological processes during Hg uptake in marine sediments, but the lack of mass-independent fractionation (MIF) renders Hg isotope systematics (especially MIF) a powerful tracer of Hg provenance^30,31^. Hg-isotopic MIF variation in Phanerozoic sedimentary successions has been interpreted in terms of source changes rather than diagenetic effects^8,15,32^.

In most of the study sections, Hg exhibits a stronger correlation to TOC (i.e., *r* ranging from+0.55 to +0.95 than to sulfur (S) (*r* mostly <+0.45) or aluminum (Al) (*r* ranging from +0.20 to +0.84 (Supplementary Fig. 12; note all *r* values significant at *p*(a) < 0.01). This strong correlation supports organic matter as the dominant Hg substrate. Although there is pronounced variation in TOC concentrations in most sections, both raw and TOC-normalized Hg concentrations (i.e., Hg/TOC) show systematic stratigraphic trends in the 10 study sections, suggesting that elevated Hg fluxes to the sediment were not simply due to increased organic matter burial. Furthermore, increases in Hg/TOC around the LPME are not related to changes in sediment lithology, as samples containing <1% Al (i.e., carbonates) and those containing >1% Al (i.e., marls and shales) show nearly identical patterns of secular Hg/TOC variation in all profiles despite paleoenvironmental differences (Supplementary Fig. 13). Thus, we infer that the large increases in Hg/TOC observed around the LPME reflect a large increase in Hg fluxes to the ocean followed by rapid Hg removal to the sediment, reflecting the short residence time of Hg in the atmosphere–ocean system.

**Fig. 4:**
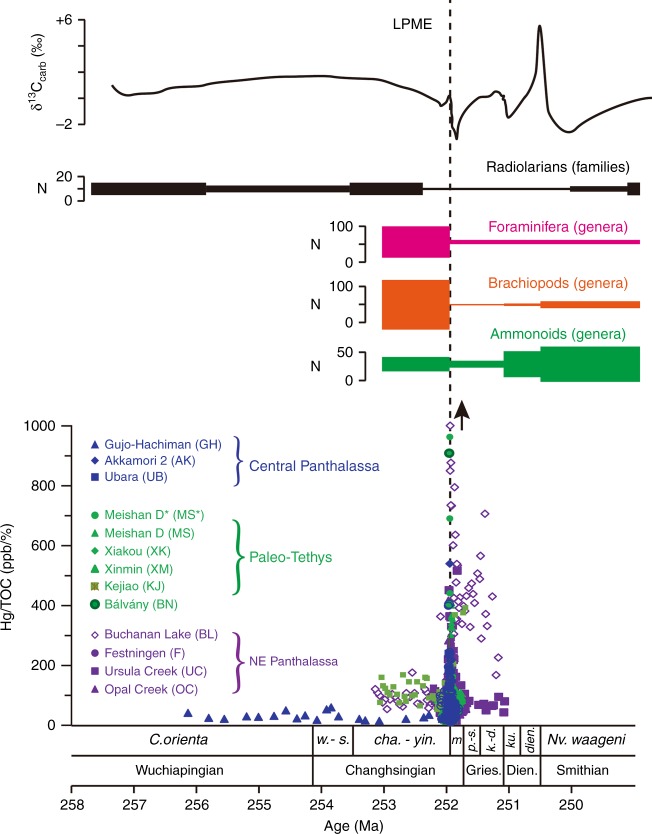
Relationship of mercury records to PTB marine ecosystem perturbations. Hg/TOC values from all study sections, biodiversity variations^70,71^, and inorganic carbon isotopes^72^. *C*. *Clarkina*, *cha*. *C. changxingensis*, *dien.* *Neospathodus dieneri*, *k.-d.* *Neoclarkina krystyni-N. discreta*, *ku*. *Sweetospathodus kummeli*, *m*. *C. meishanensis*, *Nv*. *Novispathodus*, *p.-s*. *Hindeodus parvus-Isarcicella staeschi*, *w.-s*. *C. wangi-C. subcarinata*, *yin.* *C. yini*; Gri. Griesbachian, Dien. Dienerian, LPME latest Permian mass extinction. Geochronologic and biozonation data modified from ref. ^19^, and Hg/TOC data of Buchanan Lake and Meishan D from ref. ^8^. Four samples with Hg/TOC ratios > 1000 ppb/% (Buchanan Lake = 3, Meishan D = 1) are marked by an arrow

The sharp peaks of Hg/TOC that first appear near the LPME horizon (~251.94 Ma) continue upsection in each study section for stratigraphic intervals corresponding to ~50–200 kyr. This period also corresponds to the peak of the end-Permian mass extinction, characterized by major perturbations to global biogeochemical cycles and terrestrial and marine ecosystems^1,33,34^ (Fig. 4). This timeframe is also consistent with the interval of large-scale intrusion of Siberian Traps magmas into organic-rich sediments of the Tunguska Basin during the intrusive sill-complex phase of Burgess et al. ^9^. The Hg/TOC peaks, therefore, are likely to be tied, in part, to the onset of heating of subsurface organic-rich sediments by sill intrusions of the Siberian Traps LIP rather than to the onset of flood basalt eruptions^3,9^. However, the relationship of Hg emissions to LIP activity is not well understood at present^35^.

Hg/TOC ratios exhibit only a weak relationship to distance from the Siberian Traps LIP but a strong relationship to depositional water depth (Fig. 1). Paleogeographically, sections from NE Panthalassa have higher average Hg/TOC ratios during the enrichment interval (85 ± 67 ppb/%) relative to sections from the Paleo-Tethys (62 ± 40 ppb/%) or Panthalassic oceans (30 ± 21 ppb/%; Fig. 2a, b). With regard to water depths, average Hg/TOC ratios for the pre-enrichment interval are 26 ± 14, 82 ± 60, and 27 ± 19 ppb/% for shallow, intermediate, and deep sections, respectively (Fig. 2a). Thus, intermediate-depth sections show higher background Hg/TOC values (by a factor of nearly 3) than either surface and deep-ocean sections, implying elevated aqueous Hg concentrations in the upper thermocline region (~200–500 m) of Late Permian oceans. Average EFs during the enrichment interval are 3.4 ± 0.7, 4.6 ± 1.8, and 4.9 ± 2.9 for shallow, intermediate, and deep sections, respectively (Fig. 2b), indicating that the pulse of Hg released during the PTB crisis was preferentially transferred out of the surface ocean and into deeper waters. Hg enrichment in shallow-water settings during the Toarcian (~183 Ma) was inferred to have been the result of intense terrestrial runoff^36^, although this is likely not the case for the present study sections owing to distinctly greater mercury enrichments at intermediate-depth relative to shallow-water settings. Instead, this pattern is similar to the Hg loading in the thermocline of modern oceans, which results from adsorption of Hg onto sinking organic particles and downward transfer through the biological pump^37^. However, other factors (e.g., the amount and type of organic matter) may also have influenced the depth-dependent distribution of Hg in the study sections.

There is a distinct difference in the timing of initial Hg enrichment relative to the LPME horizon in the shallow-water relative to the deep-water study sites. At shallow-water locales, the spike in Hg enrichments and faunal turnover are nearly synchronous, whereas the deep-water locales show a large time lag between the initial Hg pulse and faunal turnover. Hg/TOC peaks are ~0.5 and 0.3 m below the LPME in the deepwater Akkamori-2 and Ubara sections, representing at least a 50–100 kyr lag (Fig. 1; see Methods for age models). A smaller time gap (~20 kyr) between Hg enrichments and the LPME horizon is inferred for the intermediate-depth Xiakou section.

The synchronicity of the Hg enrichments and the extinction horizon in shallow-water sections might be related to sediment homogenization by bioturbation. However, in key sections Hg enrichments occur predominantly in sediments with limited fabric disruption^38,39^, indicating that the offsets in Hg enrichments and the extinction horizon are not linked to bioturbation. For instance, sediment homogenization at Meishan is limited to 2–4 cm just below the extinction horizon (Bed 25) and is largely lacking above the LPME^40^. The pelagic sections from Japan also exhibit strong primary sedimentary fabric preservation with only limited evidence of bioturbation^39,41^.

Mercury isotopes can be used to track the source and depositional pathways of mercury into marine sediments (see Blum et al. ^30^ and references therein) given that the two main Hg sources to the oceans, i.e., terrestrial runoff and atmospheric deposition of Hg(II), have different isotopic signatures^30,31^. Mercury has a complex biogeochemical cycle and undergoes transformations that may induce MDF (δ^202^Hg) and/or MIF (Δ^199^Hg) of Hg isotopes^30^. Volcanogenic Hg has δ^202^Hg values between ‒2‰ and 0‰^42,43^, and its MDF can be influenced by a wide range of physical, chemical, and biological processes. MIF, in contrast, occurs predominantly through photochemical processes^8,30^. Hg emitted by arc volcanoes or hydrothermal systems does not appear to have undergone significant MIF (~0‰), although a relatively limited number of settings have been studied to date. Coal combustion commonly leads to release of Hg with negative δ^202^Hg and Δ^199^Hg values^43,44^. Alternatively, photoreduction of Hg(II) complexed by reduced sulfur ligands in the photic zone can limit negative MIF^45^. However, Hg enrichments and negative MIF records in the present study units cannot be due exclusively to oceanic anoxia near the PTB, because Hg enrichments are measured in diverse redox environments and the Hg is hosted mainly by organic matter rather than sulfides.

The near-zero Δ^199^Hg values (mostly 0‰ to +0.10‰) for the pre-LPME interval at Meishan D and Xiakou may reflect photochemical reduction of Hg or the mixing of terrestrial and atmospheric sources of Hg^43^ (Fig. 3). However, the lower to middle Changhsingian interval at Gujo-Hachiman (the stratigraphic equivalents of which were not sampled in the Meishan D and Xiakou sections) exhibits distinctly elevated Δ^199^Hg compositions, ranging from +0.10‰ to +0.35‰, which are typical of marine sediments^30^ and consistent with photoreduction of aqueous Hg^II^^26,43^. All three sections (especially the pelagic Gujo-Hachiman section) exhibit near-zero, although somewhat variable, Δ^199^Hg values during and following the LPME, which are consistent with predominantly volcanic and/or thermogenic (i.e., coal-derived) Hg inputs.

MDF (δ^202^Hg) profiles for the study sections show roughly similar patterns: Meishan D and Xiakou yield background (pre-LPME and post-PTB) values of ca. ‒0.50‰, whereas the stratigraphically older part of the Gujo-Hachiman section shows more negative pre-LPME values, ranging from ‒0.80‰ to ‒2.30‰ with a mean of ‒1.50‰ (Fig. 3). All three sections show increased variability in δ^202^Hg around the LPME, with Meishan D and Xiakou each possibly displaying two negative spikes. These excursions in MDF support a change in the source or cycling of marine Hg close to the LPME, although the exact nature of the controlling processes is uncertain. For the pre-LPME interval at Gujo-Hachiman, the large positive MIF and negative MDF signatures imply a dominant atmospheric transport pathway^30,46^. The small positive MIF and negative MDF signatures of the Meishan D and Xiakou sections may indicate mixed atmospheric and terrestrial sources, with possible Hg inputs from land plants owing to increased Hg loadings in terrestrial ecosystems.

Our new Hg-isotopic results yield insights beyond those of earlier Hg studies of the PTB. Grasby et al. ^8^ inferred that δ^202^Hg-Δ^199^Hg values were consistent with Hg sourced mainly from volcanic activity for a deep slope section in the Canadian Arctic (Buchanan Lake), and a combination of atmospheric inputs and terrestrial runoff for a nearshore section in China (Meishan D). Although our minimum MIF values are much less negative than those reported by Grasby et al. ^8^, our data for Meishan D also support a mixture of terrestrial and atmospheric Hg sources. We infer that changes around the LPME in the deep-ocean Gujo-Hachiman section (near-zero to weakly positive Δ^199^Hg values, a concurrent increase of MDF, and strong Hg enrichments) are evidence of atmospheric inputs of Hg (i.e., from volcanic emissions as well as volcanic-related thermogenic sources such as coal combustion) to the open ocean thousands of kilometers distant from riverine fluxes. Overall, the trends in δ^202^Hg-Δ^199^Hg values are consistent with massive inputs of Hg from volcanic emissions and/or combustion of Hg-bearing organic-rich sediments by the Siberian Traps LIP.

The LPME coincided with the onset of sill complex formation of the Siberian Traps LIP^9^, indicating that the initial Hg enrichments near the LPME in PTB sections were also coincident with those sills. Hg profiles can provide high-resolution records of volcanic activity given the short residence time of Hg in the atmosphere and oceanic water column (<2 years and <1000 years, respectively)^37,47^. Compared to the synchronicity of Hg peaks and the LPME in shallow-water sections, the observed time gaps of ~50 to 100 kyr between the initial appearance of Hg peaks and the LPME in pelagic deep-water sections (Akkamori-2 and Ubara) may support a diachronous marine extinction event. This conclusion, however, is dependent on the geological synchronicity of the Hg peaks, which depends on the age model and the placement of the LPME in each section (see Methods). A protracted extinction model has also been proposed based on the differential timing of sponge extinctions relative to the LPME in the Arctic region^48^ and radiolarian extinctions in the Nanpanjiang Basin^49,50^.

A diachronous extinction event would provide new insights into the long-debated influence of various ‘kill mechanisms’, e.g., hypercapnia^51,52^, thermal stress^53^, and oxygen and sulfide stresses^54,55^. The effects of hypercapnia and thermal stress should be nearly synchronous, as heat and carbon dioxide are fairly evenly distributed through atmospheric and marine circulation on 1–2 kyr time scales^56^. Moreover, the effects of hypercapnia should be coincident with peak Hg enrichments and peak outgassing (assuming the two are equivalent) given that silicate and marine weathering will begin to draw down atmospheric carbon dioxide following the onset of a carbon injection (e.g., refs. ^57,58^). This is consistent with the synchronous increase in atmospheric Hg and CO_2_ during the end-Triassic crisis^15^. In contrast, ocean anoxia can develop over a wide range of time scales, depending on initial local oxygen concentrations, baseline nutrient levels, and the extent and rate of nutrient release into the marine system from enhanced weathering and positive feedbacks associated with the P cycle^59,60^. For anoxia to develop in deep-ocean settings (e.g., extensive anoxia in deep-marine settings near the LPME^24,61^), greater nutrient loading (e.g., P, Fe) is needed than for shelf settings^62^. Thus, the presence of Hg enrichment across different marine environments (assuming a volcanogenic origin) provides new evidence for oxygen stress, rather than extreme temperatures or hypercapnia, as the critical driver of Earth’s largest mass extinction event. It should also be noted that elevated temperatures reduce oxygen saturation levels in seawater and cause the metabolic effects of low oxygen to become more severe^63^.

Mercury enrichments near the LPME horizon in continental shelf, continental slope, and abyssal marine sections, combined with Hg isotopes (δ^202^Hg–Δ^199^Hg), provide evidence for a massive increase in volcanic-related Hg emissions during the Permian–Triassic biotic crisis. This study provides direct geochemical evidence from marine sections for near global-scale volcanic effects linking the Siberian Traps LIP to the PTB crisis. Relative to pre-LPME background values, Hg-EFs rose by factors of 3–8 during the mass extinction event before returning to near-background levels in the Early Triassic. Hg/TOC ratios are significantly higher (by a factor of nearly 3) in intermediate-depth sections relative to surface and deep-ocean sections prior to the PTB crisis, reflecting a general concentration of Hg within the upper thermocline region through the action of the biological pump. Further, with current placements of the LPME horizon in each section, stratigraphic differences between the initial spike of Hg concentrations and the LPME represent a time gap that provides evidence of a globally diachronous mass extinction event. Specifically, the extinction horizon in deep-water sections (e.g., Akkamori-2 and Ubara) postdated peak volcanogenic Hg inputs by ~50 to 100 kyr, whereas it was nearly synchronous in shallow-water sections. Because of feedbacks in the marine oxygen cycle, sulfide and oxygen stresses would have developed over thousands or even tens of thousands of years after the peak of volcanic outgassing. A lag between peak volcanogenic Hg inputs and biotic turnover is likely when ecosystem destabilization is caused by oxygen stress, in contrast to the geologically rapid response expected if extreme temperatures or hypercapnia were the main kill mechanism. In summary, evidence for a protracted extinction interval provides new support for oxygen and sulfide stresses as the main kill mechanism over a large swath of the ocean in response to Siberian Traps LIP volcanism.

## Methods

### Sample preparation and elemental analyses

Samples were trimmed to remove visible veins and weathered surfaces and pulverized to ~200 mesh in an agate mortar. Aliquots of each sample were prepared for different analytical procedures. Major element concentrations for the Kejiao section were determined by wavelength-dispersive X-ray fluorescence (XRF) analysis of fused glass beads using an XRF-1800 in the State Key Laboratory of Biogeology and Environmental Geology at the China University of Geosciences-Wuhan. Major element analyses of the remaining study sections had been undertaken previously in the context of other studies.

TOC concentrations for all sections except Akkamori-2 and Ubara sections were measured using an Eltra 2000 C-S analyzer at the University of Cincinnati. Data quality was monitored via multiple analyses of USGS SDO-1 standard, yielding an analytical precision (2*σ*) of ±2.5% of reported values for TOC. TOC for Akkamori-2 and Ubara was measured at Yale University using a Delta Plus. Data quality was monitored via multiple analyses of a Low Organic Content Soil Standard (B2152) and a Medium Organic Content Soil Standard (B2178), yielding a long-time (two-year) standard deviation of ±0.06%. An aliquot of each sample was digested in 2 N HCl at 50 ^o^C for 6 h to dissolve carbonate minerals, and the residue was analyzed for TOC and non-acid-volatile sulfur (NAVS); total inorganic carbon (TIC) and acid-volatile sulfur (AVS) were obtained by difference.

### Mercury concentrations and isotopes

Hg concentrations (391) were determined at the School of Earth Sciences of China University of Geosciences-Wuhan (Xinmin (32), Kejiao (36)), the Analytical and Stable Isotope Center of Yale University (Ursula Creek (37), Opal Creek (53), Bálvány (31), Akkamori-2 (59), and Ubara (28)), and the State Key Laboratory of Environmental Geochemistry, Institute of Geochemistry, Chinese Academy of Sciences, Guiyang (Meishan D (41), Xiakou (41), and Gujo-Hachiman (33)). At Yale University, Hg concentrations were analyzed for 120-mg aliquots of sample using a Direct Mercury Analyzer (DMA80). Data quality was monitored via multiple analyses of the MESS-3 standard, yielding an analytical precision (2*σ*) of ±0.5% of reported values for Hg. One replicate sample and standard were analyzed for every 10 samples. Ten replicate samples were analyzed for Hg concentrations at all three laboratories, yielding variations in reported values of less than ±5% despite some differences in methods and instrumentation.

Analysis of Hg concentrations and isotopic compositions in China followed procedures described in recent similar studies^64,65^. To ensure low blanks all teflona nd glassware were acid cleaned before use. Teflon materials including bottles and fittings were cleaned in a similar manner and air-dried for 24 h in a fume hood. We prepared the 0.2-M BrCl solution by mixing concentrated HCl with KBrO_3_ powders (>99%, ACS reagent, Aldrich, USA) at 250 °C for 12 h. We used a SnCl_2_ solution (from ACS reagent, Aldrich, USA) for Hg reduction prior to measurement of Hg concentrations by cold vapor atomic fluorescence spectroscopy (CVAFS) and Hg isotopes by MC–ICP–MS. We prepared a 0.2 g mL^−1^ NH_2_OH·HCl solution for BrCl neutralization, and the reductants were bubbled for 6 h with Hg-free N_2_ to remove trace levels of Hg.

All analyses of Hg isotopes (Meishan D (15), Xiakou (21), and Gujo-Hachiman (24)) were carried out at the State Key Laboratory of Environmental Geochemistry, Institute of Geochemistry, Chinese Academy of Sciences, Guiyang. We extracted and concentrated Hg using a previously described double-combustion and trapping dual-stage protocol^65^. We used thallium NIST SRM 997 standard (20 ng/mL Tl in 3% HNO_3_) to correct for mass bias and the international Hg standard NIST SRM 3133 to monitor analytical precision and accuracry^64,65^. We used UM-Almaden as the reference material as a secondary laboratory Hg standard (National Center for Standard Materials, Beijing, China). Ten percent of samples were duplicated—giving us a total of 20 replicate analyses. The Hg-trapping solution (a mixture of 4 M HNO_3_ and 1.3 M HCl) was diluted to a final acid concentration of ~20% and stored at 4 °C for subsequent isotope measurements. Procedural blanks were negligible (<0.13 ng, *n* = 8) relative to the amount of Hg in samples (>20 ng). We had near-complete recovery (98 ± 4%, 2 SD) guaranteeing that there was no Hg isotope fractionation during the pre-concentration procedure. Volatile ionized Hg generated by SnCl_2_ reduction in a cold-vapor generation system was introduced into the plasma (Nu-MC–ICP–MS) with Ar as a carrier gas. We used standard-sample bracketing method for all samples.

Hg isotopic results are expressed as *δ* values in units of per mille (‰) relative to the bracketed NIST 3133 Hg standard, as follows:

1 $${\mathrm{\delta }}\,{}^{202}{\mathrm{Hg = }}\left[ {\left( {\,{}^{202}{\mathrm{Hg/}}\,{}^{198}{\mathrm{Hg}}} \right)_{{\mathrm{sample}}}{\mathrm{/}}\left( {\,{}^{202}{\mathrm{Hg/}}\,{}^{198}{\mathrm{Hg}}} \right)_{{\mathrm{standard}}} - 1} \right] \times 1000\permil$$

Any Hg-isotopic value that does not follow the theoretical MDF was considered as an isotopic anomaly caused by MIF. MIF values are indicated by “capital delta (Δ)” notation (in per mille) and predicted from δ^202^Hg using the MDF law:

2 $${\mathrm{\Delta }}^{{\mathrm{199}}}{\mathrm{Hg = \delta }}^{{\mathrm{199}}}{\mathrm{Hg--0}}{\mathrm{.252}} \times {\mathrm{\delta }}^{{\mathrm{202}}}{\mathrm{Hg}}$$

3 $${\mathrm{\Delta }}^{{\mathrm{201}}}{\mathrm{Hg = \delta }}^{{\mathrm{201}}}{\mathrm{Hg--0}}{\mathrm{.752}} \times {\mathrm{\delta }}^{{\mathrm{202}}}{\mathrm{Hg}}$$

The long-term measurements of GBW07405 yielded mean values of ‒1.79 ± 0.08‰, ‒0.30 ± 0.04‰, ‒0.01 ± 0.02‰, and ‒0.28 ± 0.03‰ for δ^202^Hg, Δ^199^Hg, Δ^200^Hg, and Δ^201^Hg (2 SD, *n* = 11), respectively, in agreement with previous studies^64^. Repeated measurements (*n* = 15) of the standard UM-Almadén Hg yielded mean δ^202^Hg, Δ^199^Hg, Δ^200^Hg, and Δ^201^Hg values of ‒0.54 ± 0.11‰, ‒0.01 ± 0.03‰, 0.00 ± 0.05‰, and 0.00 ± 0.05‰ (2*σ*), respectively, also in agreement with previous studies^64,66,67^. The obtained 2*σ* value is in line with uncertainties for samples analyzed only once.

### Section correlations and time models

A high-resolution stratigraphic correlation framework among the study sections was generated on the basis of detailed biostratigraphic and chemostratigraphic data, including conodont zonations (for shallow-water carbonate settings), radiolarian zonations (for deep-water chert settings), and carbon isotope profiles. A few key features that distinguish the LPME include: (1) a lithologic change (generally toward more siliciclastic-rich compositions), (2) the first appearance of the conodont *C. meishanensis* at the base of the LPME, and (3) a pronounced negative carbon isotope excursion. The increased clay content of the beds immediately overlying the LPME has been attributed to intensification of chemical weathering on land and increased terrigenous fluxes to the ocean^68^. A ~2‰ to 6‰ negative excursion of both carbonate and organic carbon isotopes is associated with the LPME globally^69^. For the studied sections, lithological changes were obvious near the LPME, e.g., a transition from limestone to mudstone/volcanic ash beds in shallow-depth and intermediate-depth settings, and siliceous claystone to shales in the deep-water sections (Supplementary Fig. 14). For the shallow-depth and intermediate-depth sections, conodont zonations typically included the *Clarkina changxingensis changxingesis*—*C. deflecta*, *C. yini, Hindeodus praeparvus* (pre-LPME), *C. meishanensis* (LPME to PTB), *H. parvus*, *Isarcica staeschei*, and *I. isarcica* zones (post-PTB). The LPME was placed at the base of the *C. meishanensis* Zone. The deep-water sections (AK-2 and Ubara) yielded diagnostic species of radiolarians (e.g., *Albaillella* cf. *triangularis*) and conodonts (e.g., *C. changxingensis* and *C. subcarinata*) for the uppermost Permian (pre-LPME), as well as the conodont index taxon *Hindeodus parvus*, whose first appearance datum marks the base of the Triassic System (Supplementary Fig. 14). In addition, all sections yielded a well-defined negative carbon isotope excursion within the LPME-to-PTB interval (δ^13^C_carb_ for carbonate-dominated sections, and δ^13^C_org_ for chert-dominated and mudstone-dominated sections), except for Xinmin, which is characterized by numerous volcanic ashes through the boundary interval (Supplementary Fig. 14).

The timescale used in this study, which was modified from ref. ^19^, is based on a combination of radiometric dating of key stratigraphic boundaries (e.g., LPME, PTB) in each section^1,2^ and the relative durations of conodont zones. For shallow-depth and intermediate-depth sections with detailed conodont zonations, it allowed development of highly detailed age models. for the deep-water sections (Akkamori 2 and Ubara), age models were constructed on the basis of two age anchor points (251.94 Ma for the LPME and 251.90 Ma for the PTB) together with astrochronological analysis of sedimentation rates. At Akkamori 2, the 0.75 m interval between the LPME and PTB represents ~40 kyr^2^. Assuming a ~4× increase in sedimentation rates from Upper Permian cherts to Lower Triassic shales in the central Panthalassic Ocean based on astronomical cycles^68^, the time span between the onset of the Hg peak (~0.7 m below the LPME) and the LPME is thus ~100 kyr.

## Supplementary information

Supplementary Information

Source Data

## Data Availability

The authors declare that the main data supporting the findings of this study are available within the Source Data file. Extra data are available from the corresponding author upon request.
