# Supplementary material for: Evidence for a prolonged Permian–Triassic extinction interval from global marine mercury records
Source: Nat Commun. 2019 Apr 5;10:1563. doi: 10.1038/s41467-019-09620-0 (PMC6450928; doi:10.1038/s41467-019-09620-0)
Supplement: Supplementary file 1 — Supplementary Information [file 41467_2019_9620_MOESM1_ESM.pdf]

`

**Evidence for a prolonged Permian-Triassic extinction interval from global marine mercury records**

Shen et al.

**Supplementary Note 1 | Mercury cycle**

Mercury exists in three main forms in the atmosphere: (1) gaseous elemental Hg ($Hg^0$), (2) atmospheric $Hg^{2+}$ compounds that are commonly associated with particles ($Hg_P$), and (3) halide compounds (referred to collectively as reactive gaseous mercury, RGM)[1]. Hg is distributed globally mainly in the form of $Hg^0$, which compromises 90% of total atmospheric Hg and has an atmospheric residence time of ~6 months to 1 year, allowing for long-distance transport[2,3]. $Hg^0$ can be oxidized to $Hg^{2+}$, which is removed from the atmosphere through both wet (RGM) and dry ($Hg_P$) deposition[3,4,5]. $Hg^{2+}$ can be methylated into neurotoxic and bioaccumulative methylmercury (MMHg) in the aqueous environment[6,7], which poses a serious threat to human health via fish or rice consumption[8,9]. In addition to atmospheric deposition, Hg is also delivered to the ocean by river-borne particles (e.g., clays and organic matter)[10,11].

In the aqueous environment, Hg is present mainly as elemental mercury ($Hg^0_{aq}$), divalent inorganic mercury ($Hg^{2+}_{aq}$), monomethylmercury (MMHg), dimethylmercury (DMHg), and particle-bound mercury ($Hg^P_{aq}$)[12]. Hg has somewhat variable behavior with water depth in the ocean[13]. In the surface layer, $Hg^{2+}_{aq}$ can be reduced to $Hg^0_{aq}$ and then reemitted to the atmosphere. $Hg^{2+}_{aq}$ is also adsorbed onto suspended organic particulates (= $Hg^P_{aq}$) in amounts that are generally proportional to primary production. Most $Hg^P_{aq}$ is released back to the water column during remineralization of organic matter, leading to increased concentrations of $Hg^{2+}_{aq}$ within the oceanic thermocline region. Methylation of $Hg^{2+}_{aq}$ to MMHg and DMHg occurs mainly in oxygen-minimum zones (OMZs). Only a small fraction of $Hg^P_{aq}$ reaches the deep-ocean floor to accumulate in abyssal deposits. In marine sediments, Hg tends to form strong, stable complexes with organic matter and/or HgS minerals and Hg-S complexes that resist remobilization in the burial environment[14].

The large reservoir of Hg in the ocean (~953 × $10^6$ mol) plays an important role in the Earth's Hg cycle[13]. In the open ocean, Hg concentrations are mainly between 0.1

and 0.2 pmol in the surface mixed layer, yielding an inventory of ~$3 \times 10^6$ mol Hg[13]. The intermediate layer (~200–1000 m) often has the higher Hg concentrations (~0.4 pmol) due to remineralization of sinking organic particulates, yielding an inventory of $110 \times 10^6$ mol Hg. Hg concentrations increase slightly downward to the abyssal seafloor (~0.8 pmol), yielding an inventory in the deep ocean of $840 \times 10^6$ mol Hg. The residence times of Hg in the deep ocean (1700 yr), and intermediate layer (120 yr) are much longer than that in the surface mixed layer (7 months) and atmosphere (6–12 months)[13].

Hg has seven stable isotopes (196, 198, 199, 200, 201, 202 and 204) with a relative mass span of ~4 %[15]. Hg isotopes can be used to trace the sources and pathways of Hg in various environments (see Blum *et al.*[16], and references therein). Recent studies have discovered both mass-dependent fractionation (MDF) and mass-independent fractionation (MIF) of Hg isotopes in natural samples (see refers.15,16,17, and references therein). The MDF results from differences in the zero-point energies of various isotopes, and can occur during various physical, chemical and biological processes[18]. The MIF fractionation mainly caused by the nuclear volume effect (NVE) and magnetic isotope effect (MIE), and can be used to explore specific processes such as $Hg^0$ volatilization, dark Hg(II) reduction, and photochemical processes (see refers.15, 16, and references therein). Nearly all kinetic reactions (e.g., natural sunlight with fulvic acid, dark organically mediated reduction, chemical reduction, photolysis with formic acid, ethylation, volatilization) involving Hg produce products with lower $\delta^{202}$Hg and leave a residual pool of reactant with higher $\delta^{202}$Hg. Biotic and dark abiotic reactions do not produce significant MIF (i.e., $^{199}$Hg). In contrast, all photochemical reactions that have been studied produce changes in both MDF and MIF (see refers.15, 16, and references therein). The largest positive MIF of Hg isotope is caused by photochemical degradation of methylmercury in water, and the largest negative MIF of Hg isotopes is caused by photochemical reduction of inorganic Hg.

**Supplementary Note 2 | Study sections**

`

This study examined eleven sections with a near-global distribution. Two sections are from the NE Panthalassic Ocean (or NW Pangean margin), five from the Paleo-Tethys ocean, and three from the central Panthalassic Ocean (Supplementary Fig. 1).

***NE Panthalassic Ocean***

*Opal Creek, Kananaskis Country, Alberta, western Canada:* The Opal Creek section (51.0763 °N, 115.1288 °W) was deposited on the western margin of Pangea in a deep-shelf to slope setting at water depths of ~200–500 m[19] (Figs. 1 and Supplementary Fig. 1). The Lower and Middle Permian are represented by the Johnston Canyon Formation and Ranger Canyon Formation, comprising cherts deposited in a productive continent-margin upwelling system[20]. The Upper Permian is represented by a thin (~1 m), highly condensed interval of chert and siltstone at the base of the Sulphur Mountain Formation, which is assigned to the *M. sheni* and *C. hauschkei-C. meishanensis* zones of late Changhsingian age. The Lower Triassic consists of ~42 m of organic-rich silty shales and siltstones of the Sulphur Mountain Formation assigned to the *H. parvus-C. taylorae* and *C. taylorae-C. cf. carinata* zones of Griesbachian age[20] (Supplementary Fig. 2). This section has been the subject of several biostratigraphic and chemostratigraphic studies[19,20,21,22,23,24,25].

*Ursula Creek, Lake Williston, British Columbia, western Canada:* The Ursula Creek section (56.0144 °N, 123.9704 °W) was deposited on the western margin of Pangea in a deep-shelf to slope setting at water depths of ~200–500 m. The Upper Permian consists mainly of cherts of the Fantasque Formation, overlain by uppermost Permian to Lower Triassic shales (with a few sandy shales and chert interbeds) belonging to the Grayling and Toad formations[26]. The recovery of *M. sheni* from the uppermost chert beds of the Fantasque Formation and *H. parvus* from ~80 cm above the base of the Grayling Formation constrains placement of the latest Permian mass extinction (LPME) horizon and the Permian-Triassic boundary (PTB) to near the formation boundary (Supplementary Fig. 3). The Grayling Formation ranges from Griesbachian to mid-Dienerian in age, and the Toad Formation spans the mid-Dienerian to end-Spathian or possibly even younger[26]. The microfacies[26] and

`

geochemistry[21,22,27,28] of this section are well-studied.

### *Paleo-Tethys Ocean*

Four sections are from South China, which was a small craton located in the eastern part of the Paleo-Tethys near the equator during the Permian-Triassic transition (Fig. 1). Many marine PTB successions in the South China region provide a continuous record of sedimentation representing transitional facies (Zhejiang-Fujian-Guangdong clastic region), shallow carbonates (Yangtze carbonate platform), and biogenic silicates and cherts (in deepwater basins)[29,30,31]. The Northern Marginal and Nanpanjiang deepwater basins, located to the north and south of the Yangtze Platform, respectively, are characterized by more continuous sedimentation through the Upper Permian-Lower Triassic interval than correlative shallow-water sections owing to a major regression close to the PTB[31]. The Nanpanjiang Basin contains mainly biosiliceous deposits, whereas the Northern Marginal Basin contains both shallow and deepwater carbonate deposits (the Changxing and Dalong formations, respectively. All of these sections have yielded abundant conodont elements, facilitating correlations and dating of PTB sections across the South China Craton. Indeed, several important boundaries are defined based on South Chinese sections, including placement of the Permian-Triassic system boundary at the base of the *H. parvus* Zone at Meishan D[32]. In this study, we chose one shallow-water section (Meishan D) and three deep-water sections (Xiakou, northern basin; Xinmin and Kejiao, southern basin) (Fig. 1B).

*Meishan D, Zhejiang Province, South China:* The Meishan D section (31.0831 °N, 119.7088 °E) was located in an upper-shelf setting at water depths of 30-50 m. It is among the best-studied PTB sections globally owing to its status as the Global Stratotype Section and Point (GSSP) for the PTB[32]. The Upper Permian consists mainly of fossil-rich limestones of the Changxing Formation. Fossil-poor argillaceous limestones of the Yinkeng Formation dominate the lowermost Triassic, interbedded with some volcanic ash and mudstone beds in the lower part of the unit. High-resolution conodont zones were established through the Permian-Triassic

`

transition interval (from Bed 22 to Bed 32)[32] (Supplementary Fig. 4). Numerous studies have investigated the Meishan D section with regard to its fossil biotas[33,34], geochronology[35,36], geochemistry[21,22,37], biomarker content[38,39,40,41,42], and integrated history[43].

*Xiakou, Hubei Province, South China:* The Xiakou section (31.3428 °N, 111.8614 °E) was deposited on the northern margin of the South China Craton in a deep-shelf setting at water depths of ~200–300 m[44]. It consists mainly of black shale and volcanic ash interbeds in the uppermost Permian, and muddy carbonates with volcanic ash interbeds in the lowermost Triassic[45,46,47] (Supplementary Fig. 5). It was chosen for this study because it (1) records more continuous sedimentation across the PTB than shallow-water sections[31], (2) has a well-developed high-resolution conodont zonation[44,48], and (3) is well-studied with respect to its paleobiology and geochemistry[24,47,49,50,51].

*Xinmin, Guizhou Province, South China:* The Xinmin section (26.3454 °N, 105.9316 °E) is located within the Nanpanjiang Basin, on the southern margin of the South China Craton. It was deposited in a deep-shelf setting at water depths of ~200-500 m[46] (Fig. 2A). The upper Changhsingian (~8 m) consists of siliceous mudstone and limestone of the Dalong Formation, which has yielded conodonts of the *C. changxingensis* and *C. meishanensis* zones. The lower Griesbachian (~3 m) consists of mudstone of the Daye Formation, containing only a few bivalves[46] (Supplementary Fig. 6). Numerous volcanic ash beds are found throughout the section[45,46,52].

*Kejiao, Guizhou Province, South China:* The Kejiao section (25.8460 °N, 106.5870 °E) is a newly discovered PTB outcrop section in Huishui County, ~50 km south of Guiyang. It was deposited in a deep-shelf setting at water depths of ~200–500 m, similar to the Xinmin section. It consists mainly of black mudstone and shale with numerous volcanic ash interbeds in the Upper Permian Dalong Formation, and yellow mudstone containing few fossils in the Lower Triassic Daye Formation. The LPME coincides with the Dalong-Daye formation contact, as in other sections across the Nanpanjiang Basin (Supplementary Fig. 7).

*Bálvány, Bűkk Mountains, northern Hungary:* The Bálvány section was located

in the western Paleo-Tethys Ocean[53,54]. This section represents a composite of two outcrop, Bálvány North (48.0964 °N, 20.4717 °E) and Bálvány East (48.1028 °N, 20.4747 °E), which are separated from each other by ~500 m. It was deposited in a deep-shelf setting below storm wavebase[53]. The base of the Bálvány North section consists of black bituminous limestones and thin intercalated nodular marls of the Nagyvi Formation that have been assigned a latest Permian age based on occurrences of the conodont *H. praeparvus*[54]. The overlying unit is a ~1-m-thick shaly interval known as the 'Boundary Shale Beds' (BSB), which is followed by limestones of the lowermost Triassic Gerennavár Formation. The LPME and PTB have been placed at the bases of the BSB and the Gerennavár Formation, respectively[54,55]. The Bálvány East section consists mainly of platy stromatolitic limestones in its lower part and thick-bedded mudstones in its upper part, all belonging to the Gerennavár Formation[56] (Supplementary Fig. 8).

***Central Panthalassic Ocean***

The Panthalassic Ocean covered >50% of Earth's surface and was nine times larger than the Tethys Oceans during the Triassic, yet paleoenvironmental conditions within it remain very poorly known owing mainly to the fact that most of its oceanic crust and sediments have been subducted[57,58]. A few slivers of deep-ocean sediments obducted onto the Japanese microcontinent provide the best records of conditions in the central Panthalassic Ocean.

*Gujo-Hachiman, central Japan:* The Gujo-Hachiman section (35.7355 °N and 136.8489 °E) was located in the central Panthalassic Ocean (Fig. 1), accumulating a thin section of abyssal seafloor sediments at ~5000 m water depth (refers.[59,60], and references therein). The section comprises 6.9 m of green-gray ribbon chert of biosiliceous origin belonging to the Wuchiapingian *Neoalbaillella optima-Albaillella lauta* Zone and Changhsingian *A. angusta-A. flexa*, *A. triangularis*, *A. yaoi*, and *A. degradans* zones[61,62], overlain by 0.6 m of black shale of Griesbachian age[59,63] (Supplementary Fig. 9). Previous geochemical studies were focused mainly on productivity and redox changes (e.g., refers 21,22,24,59). The present study is the first

`

to analyze Hg variations in a PTB deep-ocean section.

*Akkamori 2, central Japan:* The Akkamori 2 section (39.8430 °N, 141.7963 °E) is located northwest of the town of Iwaizumi in Iwate Prefecture, northeastern Japan. It accumulated in an abyssal deep-ocean setting during the Permian-Triassic transition (Fig. 1; refer. 64, and references therein). It consists of bedded chert (Upper Permian), siliceous claystone (uppermost Permian), and carbonaceous black shale (uppermost Permian to lowermost Triassic)[65]. The index fossils *Albaillella triangularis* (Radiolaria) and *Hindeodus parvus* (Conodonta) were recovered in the siliceous claystone and the black shale, respectively. The LPME was placed near the contact of the siliceous claystone and carbonaceous shale, corresponding to an abrupt decrease in microfossil abundance and a negative organic carbon isotope excursion. The PTB was placed ~75 cm above the LPME based on the lowest occurrence of a *Hindeodus parvus* specimen[65].

*Ubara, central Japan:* The Ubara section (35.189896 °N, 135.2515 °E) is located near the village of Ubara in the Ayabe area (Kyoto Prefecture) of central Japan[66]. Its lithologic succession is similar to those of Gujo-Hachiman and Akkamori 2. The Upper Permian consists of ~1.2 m of gray cherts, overlain by ~0.4 m of siliceous claystone of latest Permian age and ~1.0 m of black shale of mostly earliest Triassic age. The LPME was placed near the contact of the siliceous claystone and black shale based on the presence of a pronounced negative organic isotope excursion[67].

`

Supplementary Figure 1 summarizes the range and depositional environments of the eleven study sections. Supplementary Figures 2 to 11 provide the raw geochemical data for these sections. Supplementary Figure 12 shows examples of the relationships of Hg to TOC, reduced sulfur, and clay minerals in two study sections (Ursula Creek and Xiakou). Supplementary Figure 13 shows the Hg/TOC data as a function of lithology, dividing samples into two subsets by Al content: those with <1 % Al being clean carbonates, and those with >1% Al being marls and shales. This figure demonstrates that the Hg/TOC results are not a function of lithology, as the Hg/TOC patterns of the two sample subsets are indistinguishable. Supplementary Figure 14 show the correlations among the eight sections (Kejiao and Gujo-hachiman are absent for the lacking carbon isotope data).

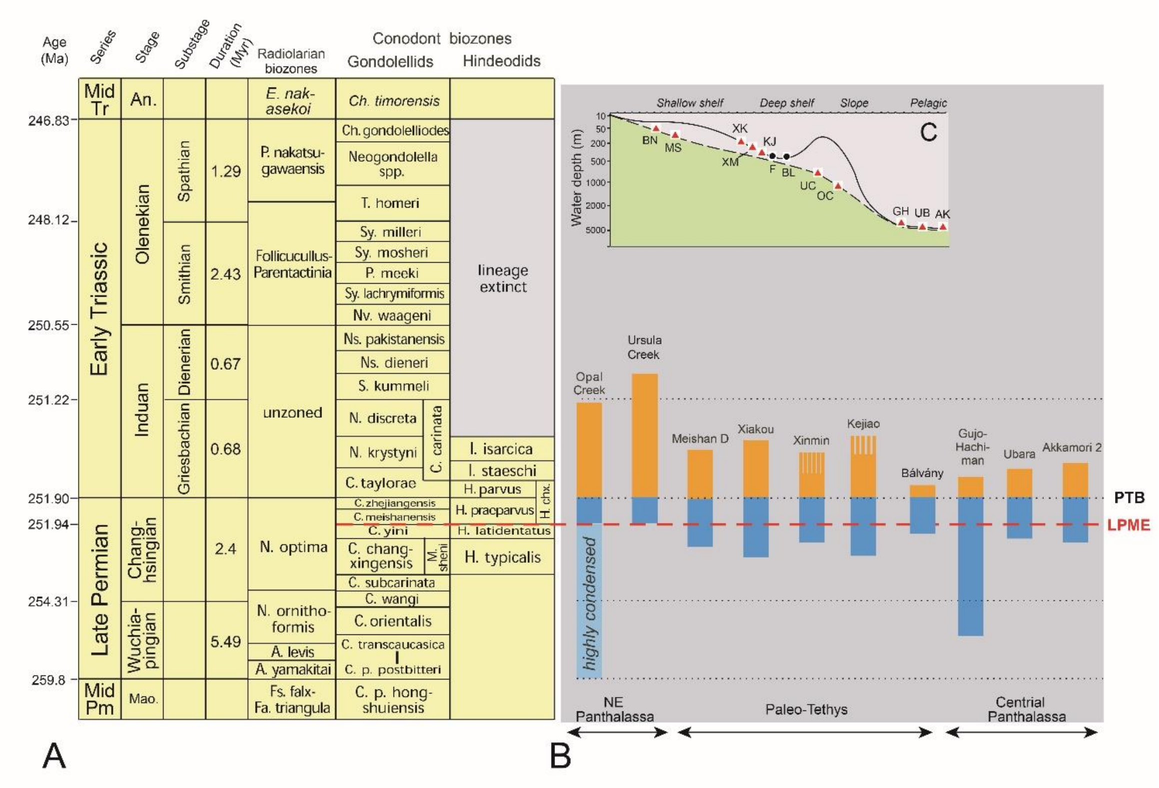


Supplementary Figure 1. (A) Timescale and biostratigraphic zonation for the Late Permian-Early Triassic (modified from Shen *et al.*[24]). (B) Stratigraphic ranges of the study sections (vertical stripes indicate uncertain age ranges). Abbreviations: radiolarian genera: *A.* = *Albaillella*, *E.* = *Eptingium*, *Fa.* = *Foremanhelena*, *Fs.* = *Follocucullus*, *N.* = *Neoalbaillella*, *P.* = *Parentactinia*; conodont genera: *C.* = *Clarkina*, *Ch.* = *Chiosella*, *H.* = *Hindeodus*, *I.* = *Isarcicella*, *M.* = *Mesogondolella*, *N.* = *Neoclarkina*, *Ns.* = *Neospathodus*, *Nv.* = *Novispathodus*, *P.* = *Paulella*, *S.* = *Sweetospathodus*, *Sy.* = *Scythogondolella*, *T.* = *Triassospathodus*. (C) Depositional settings of study sections (refer.[24], and references therein). Study sections (red triangles): AK = Akkamori 2; BN = Bálvány; BL = Buchanan Lake; F = Festningen; GH = Gujo-Hachiman; KJ = Kejiao; M = Mud; MS = Meishan; OC = Opal Creek; UB = Ubara; UC = Ursula Creek; XK = Xiakou; XM = Xinmin. Black circles represent sections with previously published Hg data[70,71].

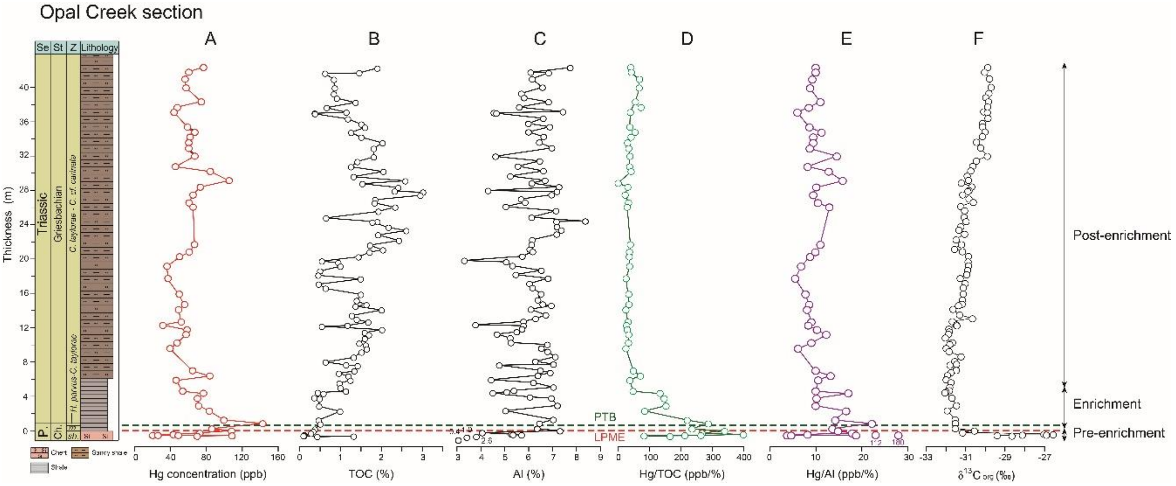


Supplementary Figure 2. Profiles of Opal Creek section: (A) Hg concentration (ppb); (B) Total organic carbon concentration (%); (C) Aluminum concentration (%); (D) ratio of Hg to total organic carbon Hg/TOC (ppb/%); (E) ratio of mercury to aluminum Hg/Al (ppb/%); and (F) organic carbon isotope $\delta^{13}C_{org}$ (‰). *m.* = *C. meishanensis* Zone, sh. = *Mesogondolella sheni* Zone; Ch. = Changhsingian, P. = Permian; Se = Series, St = Stage, Z = conodont3 zone. LPME = Latest Permian mass extinction, PTB = Permian-Triassic boundary. Refer to the main text for the definition of Pre-enrichment, Enrichment and Post-enrichment intervals. Source data are provided as a Source Data file.

`

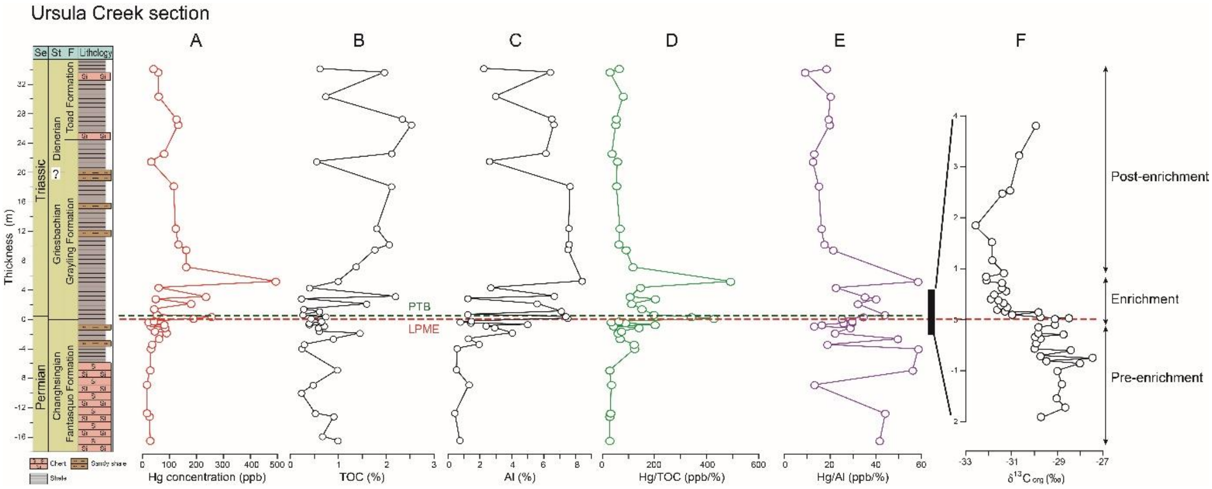


Supplementary Figure 3. Profiles of Ursula Creek section: (A) Hg concentration (ppb); (B) Total organic carbon concentration (%); (C) Aluminum concentration (%); (D) ratio of mercury to total organic carbon Hg/TOC (ppb/%); (E) ratio of mercury to aluminum Hg/Al (ppb/%); and (F) organic carbon isotope $\delta^{13}C_{org}$ (‰). Se. = Series, St = Stage, F = formation; LPME = Latest Permian mass extinction, PTB = Permian-Triassic boundary. Other details as in Figure 2. Source data are provided as a Source Data file.

`

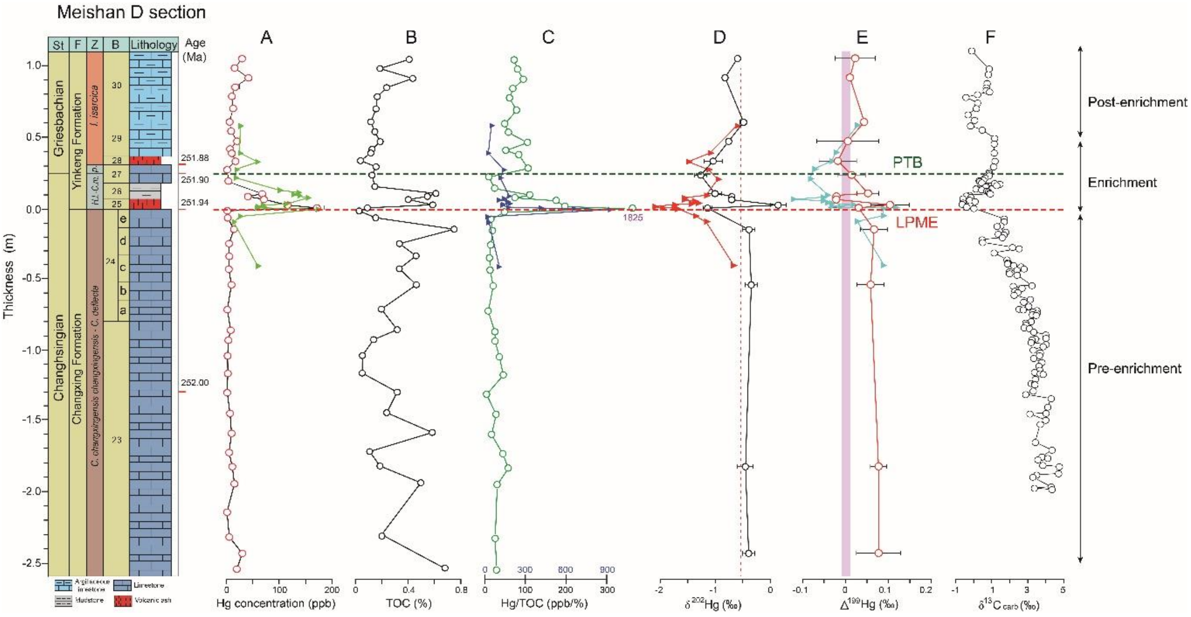


Supplementary Figure 4. Profiles of Meishan D section: (A) Hg concentration (ppb); (B) Total organic carbon concentration (%); (C) ratio of mercury to total organic carbon Hg/TOC (ppb/%); (D) mass-dependent fractionation ($\delta^{202}$Hg); (E) mass-independent fractionation ($\Delta^{199}$Hg); and (F) carbonate carbon isotope $\delta^{13}C_{carb}$ (‰). The ages are based on radiometric data[35,69]. The red dashed line in column D indicates the average background $\delta^{202}$Hg value of –0.65 ‰. The light fuchsia rectangle in column E shows the 0-0.10 ‰ range of $\Delta^{199}$Hg. The triangles in column B, C, and D and the blue scale (in column B) are data from refers[71,72]. Other details as in Figure 2. Source data are provided as a Source Data file.

`

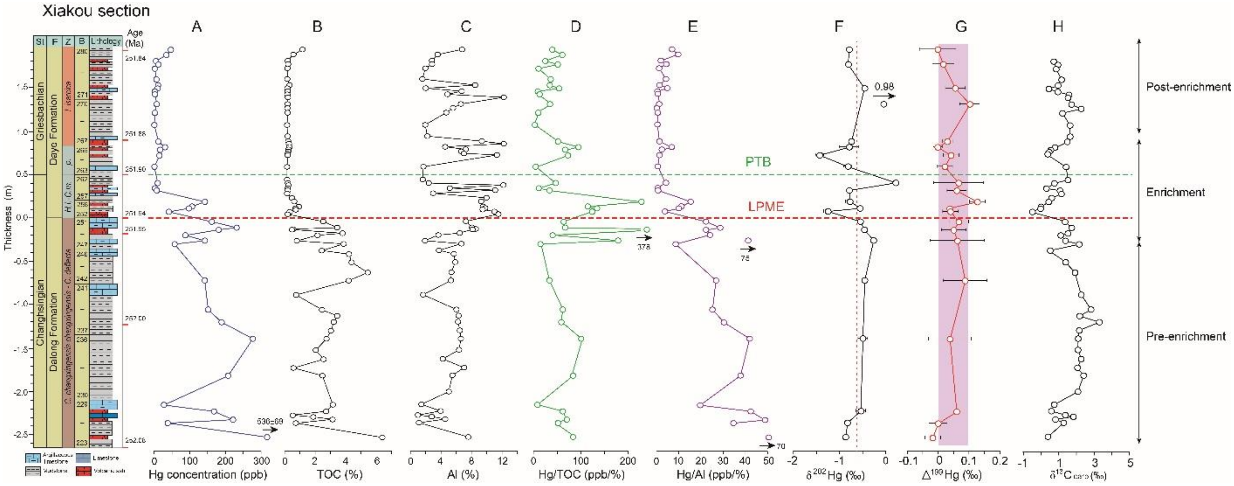


Supplementary Figure 5. Profiles of Xiakou section: (A) Hg concentration (ppb); (B) Total organic carbon concentration (%); (C) Aluminum concentration (%); (D) ratio of mercury to total organic carbon Hg/TOC (ppb/%); (E) ratio of mercury to aluminum Hg/Al (ppb/%); (F) mass-dependent fractionation ($\delta^{202}$Hg); (G) mass-independent fractionation ($\Delta^{199}$Hg); and (H) carbonate carbon isotope $\delta^{13}C_{carb}$ (‰). The 1$^{st}$ and 2$^{nd}$ extinctions are equivalent to Meishan D Beds 25 and 28, respectively. The ages are based on radiometric data[35,69]. The red dashed line in column D indicates the average background $\delta^{202}$Hg value of –0.65 ‰. The light fuchsia rectangle in column E shows the 0-0.10 ‰ range of $\Delta^{199}$Hg. Other details as in Figure 2. Source data are provided as a Source Data file.

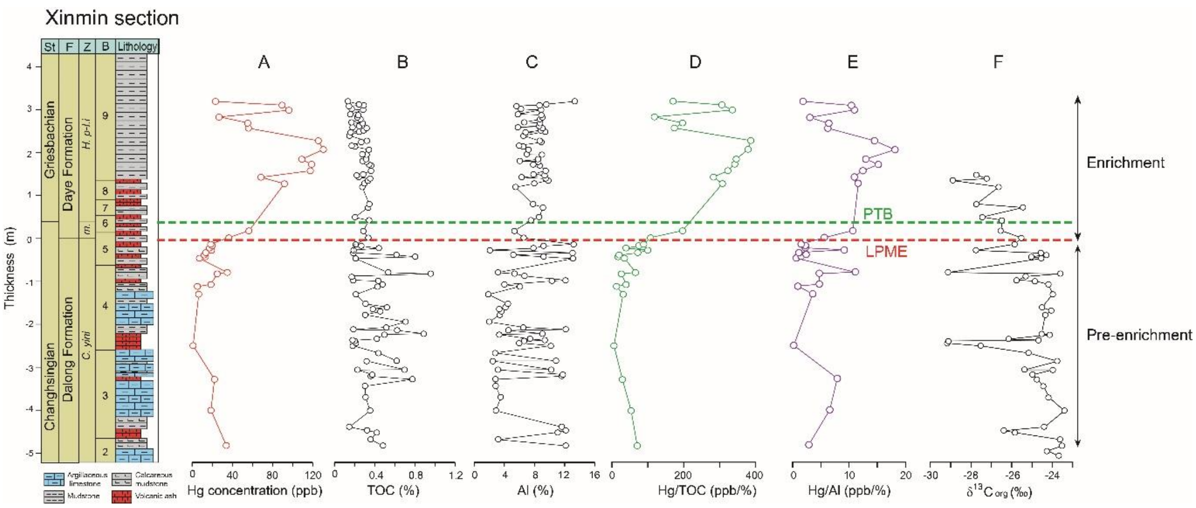


Supplementary Figure 6. Profiles of Xinmin section: (A) Hg concentration (ppb); (B) Total organic carbon concentration (%); (C) Aluminum concentration (%); (D) ratio of mercury to total organic carbon Hg/TOC (ppb/%); (E) ratio of mercury to aluminum Hg/Al (ppb/%); and (F) organic carbon isotope $\delta^{13}C_{org}$ (‰). *C.* = *Clarkina*, *H. p-I.i* = *H. parvus-I.isarcica Zone, m.* = *C. meishanensis Zone.* St = Stage, F = formation, Z = conodont zone, B = bed; LPME = Latest Permian mass extinction, PTB = Permian-Triassic boundary. Other details as in Figure 2. Source data are provided as a Source Data file.

`

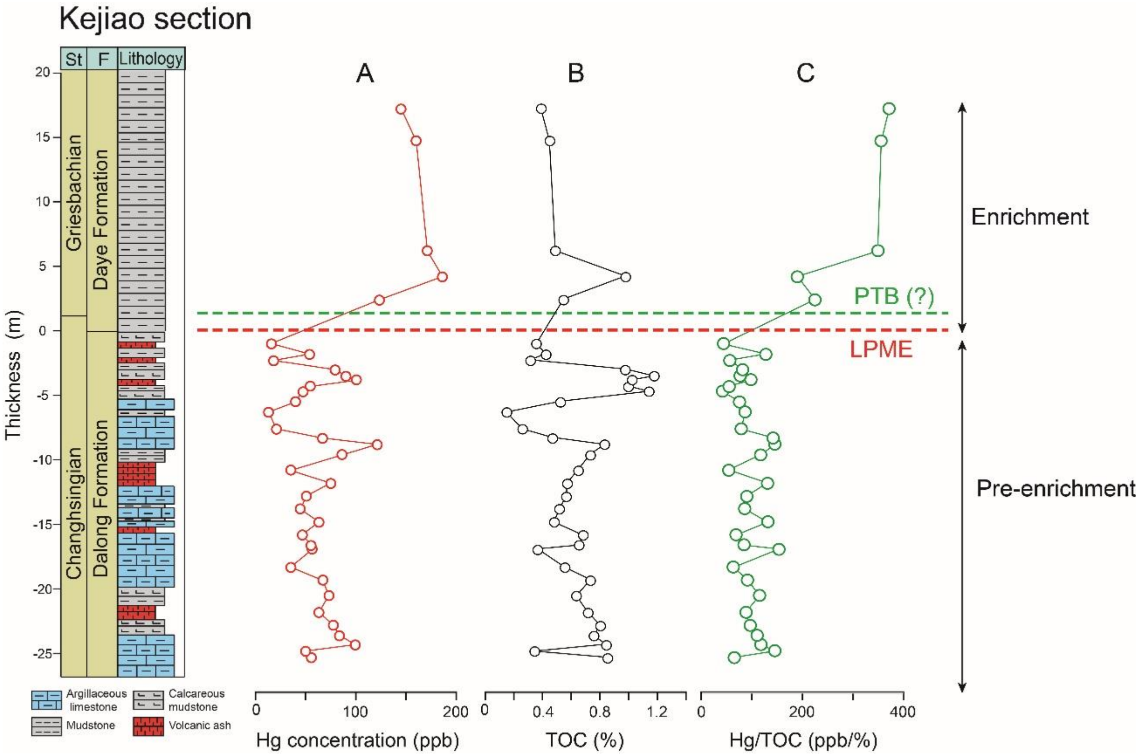


Supplementary Figure 7. Profiles of Kejiao section: (A) Hg concentration (ppb); (B) Total organic carbon concentration (%); and (C) ratio of mercury to total organic carbon Hg/TOC (ppb/%). St = Stage, F = formation; LPME = Latest Permian mass extinction, PTB = Permian-Triassic boundary. Other details as in Figure 2. Source data are provided as a Source Data file.

`

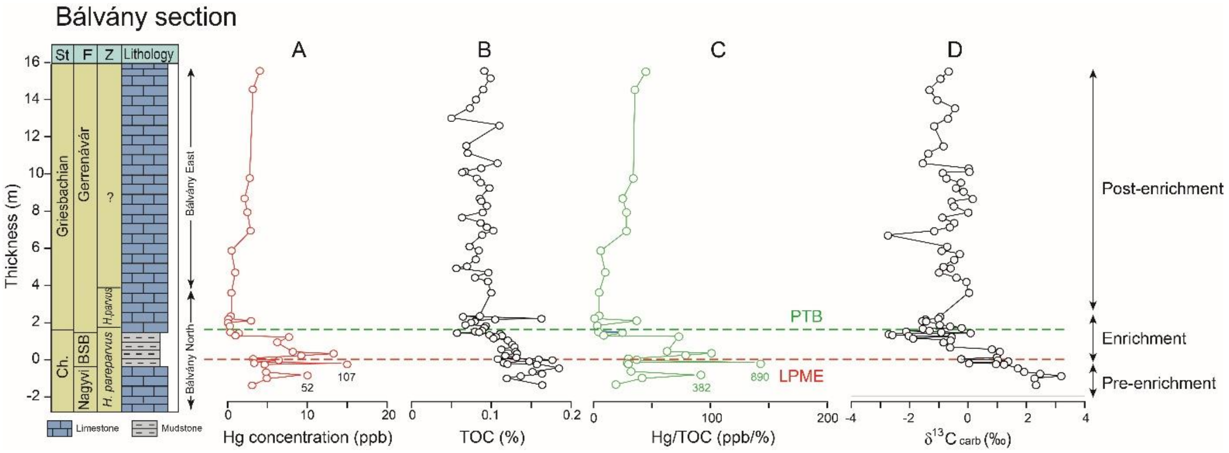


Supplementary Figure 8. Profiles of Bálvány section: (A) Hg concentration (ppb); (B) Total organic carbon concentration (%); (C) ratio of mercury to total organic carbon Hg/TOC (ppb/%); and (D) carbonate carbon isotope $\delta^{13}C_{carb}$ (‰). Ch. = Changhsingian, St = Stage, F = formation; LPME = Latest Permian mass extinction, PTB = Permian-Triassic boundary. Other details as in Figure 2. Source data are provided as a Source Data file.

`

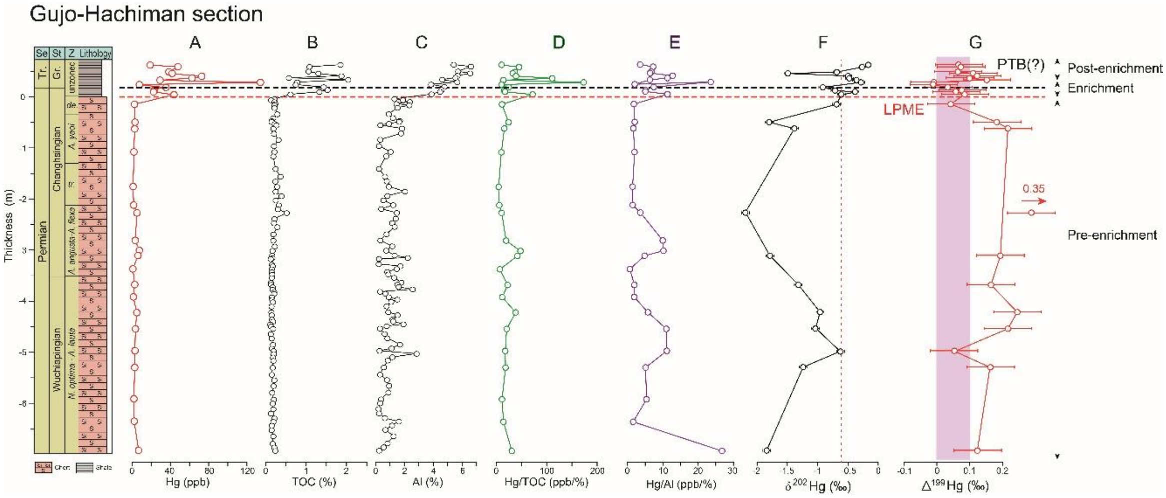


Supplementary Figure 9. Profiles of Gujo-Hachiman section: (A) Hg concentration (ppb); (B) Total organic carbon concentration (%); (C) Aluminum concentration (%); (D) ratio of mercury to total organic carbon Hg/TOC (ppb/%); and (E) ratio of mercury to aluminum Hg/Al (ppb/%); (F) mass-dependent fractionation ($\delta^{202}$Hg); and (G) mass-independent fractionation ($\Delta^{199}$Hg). The green dashed line in column D indicates the average background $\delta^{202}$Hg value of –0.65 ‰. The light fuchsia rectangle in column E shows the 0-0.10 ‰ range of $\Delta^{199}$Hg (‰). A. = *Albaillella*, de. = *A. degradans*, N. = *Neoalbaillella*, tr. = *A. triangularis*; Gr. = Griesbachian, Tr. = Triassic, Se = Series, St = Stage, Z = radiolarian zones, LPME = Latest Permian mass extinction, PTB = Permian-Triassic boundary. Other details as in Figure 2. Source data are provided as a Source Data file.

`

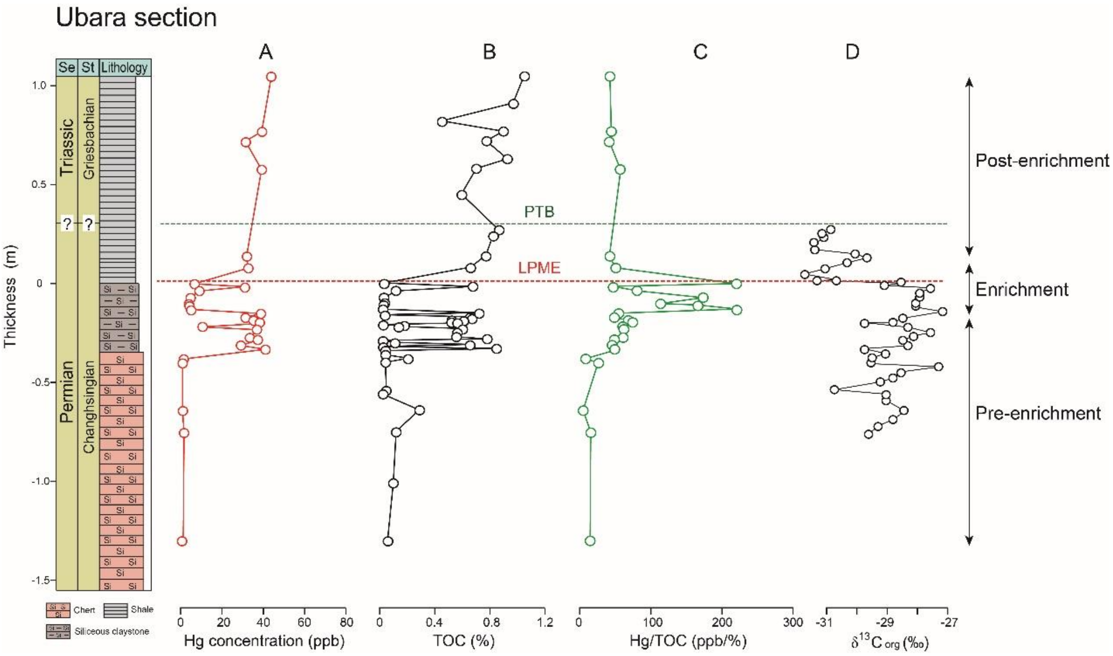


Supplementary Figure 10. Profiles of Ubara section: (A) Hg concentration (ppb); (B) Total organic carbon concentration (%); (C) ratio of mercury to total organic carbon Hg/TOC (ppb/%); and (D) organic carbon isotope $\delta^{13}C_{org}$ (‰). Se = Series, St = Stage, LPME = Latest Permian mass extinction, PTB = Permian-Triassic boundary. Other details as in Figure 2. Source data are provided as a Source Data file.

`

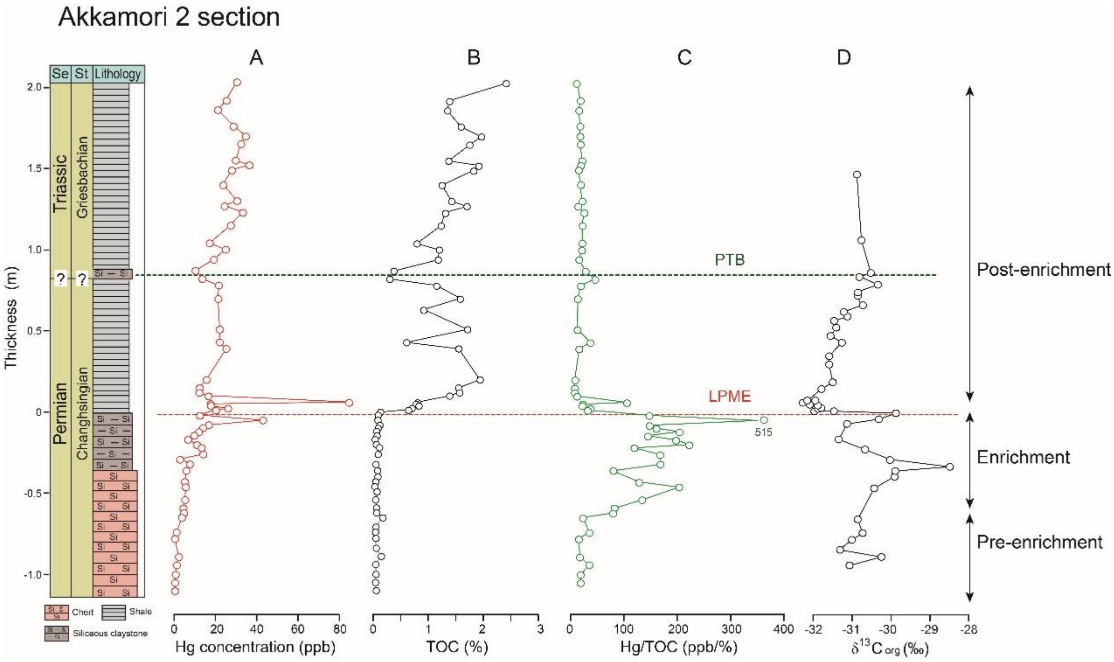


Supplementary Figure 11. Profiles of Akkamori 2 section: (A) Hg concentration (ppb); (B) Total organic carbon concentration (%); (C) ratio of mercury to total organic carbon Hg/TOC (ppb/%); and (D) organic carbon isotope $\delta^{13}C_{org}$ (‰). Se = Series, St = Stage, LPME = Latest Permian mass extinction, PTB = Permian-Triassic boundary. Other details as in Figure 2. Source data are provided as a Source Data file.

`

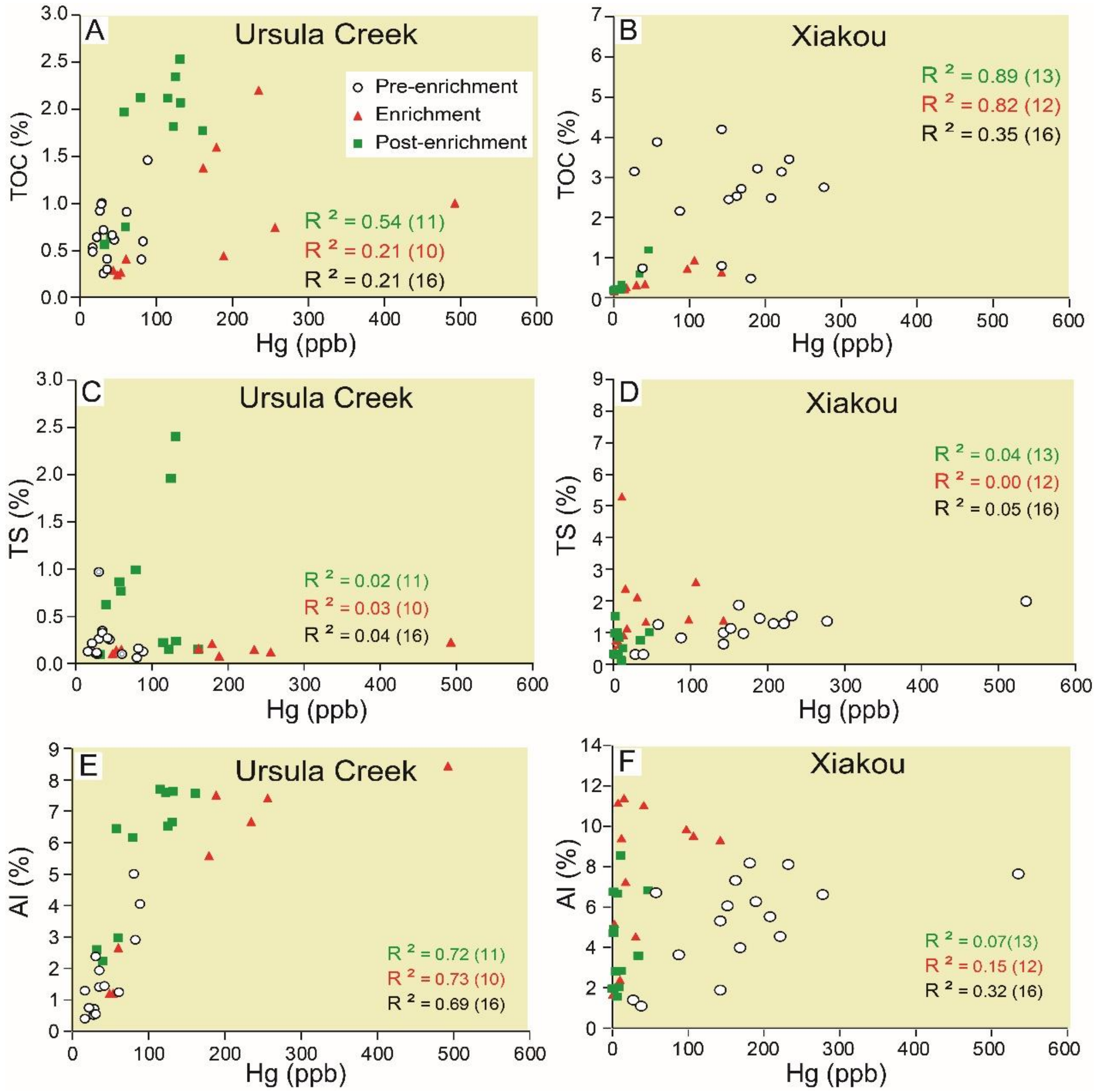


Supplementary Figure 12. Total organic carbon (TOC) versus Hg concentration (A and B), total sulfur (TS) versus Hg concentration (C and D), and Aluminum concentration (Al) versus Hg concentration (E and F) in pre-enrichment (open circles), enrichment (red triangles), and post-enrichment intervals (green rectangles) for the Ursula Creek and Xiakou sections. Black, red, and green $R^2$ are the coefficients of determination for these three intervals, respectively, and the numbers in brackets indicate the number of samples in each interval. Source data are provided as a Source Data file.

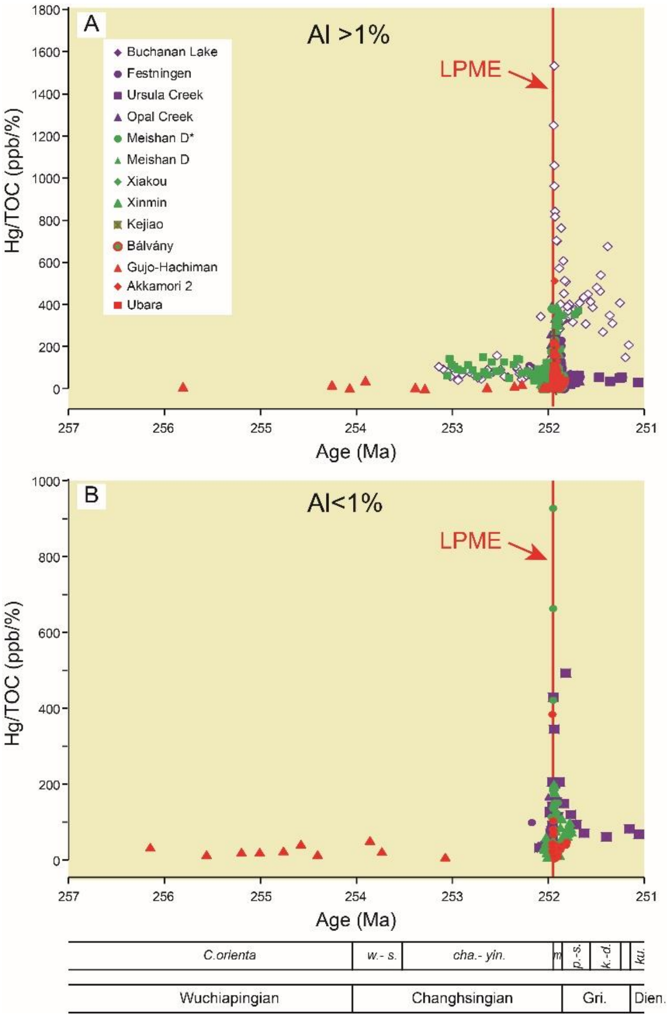


Supplementary Figure 13. Hg/TOC values of study sections having (A) Al concentrations >1 %, and (B) Al concentrations <1 %. Meishan D δ$^{13}$C profile from Burgess and Bowring (2015)[36], and Buchanan Lake, Festningen, and Meishan D* data from Grasby *et al.*[71,73]. *cha.* = *C. changxingensis*, *dien.*= *Ns. dieneri*, *dis.*= *N. discreta, k.-d.*= *N. krystyni-N. discreta*, *ku.*= *S.kummeli*, *m.* = *C. meishanensis*, *p.-h.*= *Ns. pingdingshanensis-T. homeri*, *p.-s.*= *H. parvus-I. staeschi*, *w.*= *Nv. waageni*, *w.-s.*= *C. wangi-C. subcarinata*, *yin.* = *C. yini*; Dien. = Dienerian, Gr. = Griesbachian, Sm. = Smithian. LPME = Latest Permian mass extinction. Other details as in Figure 4. Note, the date from both of Akkamori 2 and Ubara sections were assumed as Al concentrations >1% for lack of Al data for these samples. Source data are provided as a Source Data file.

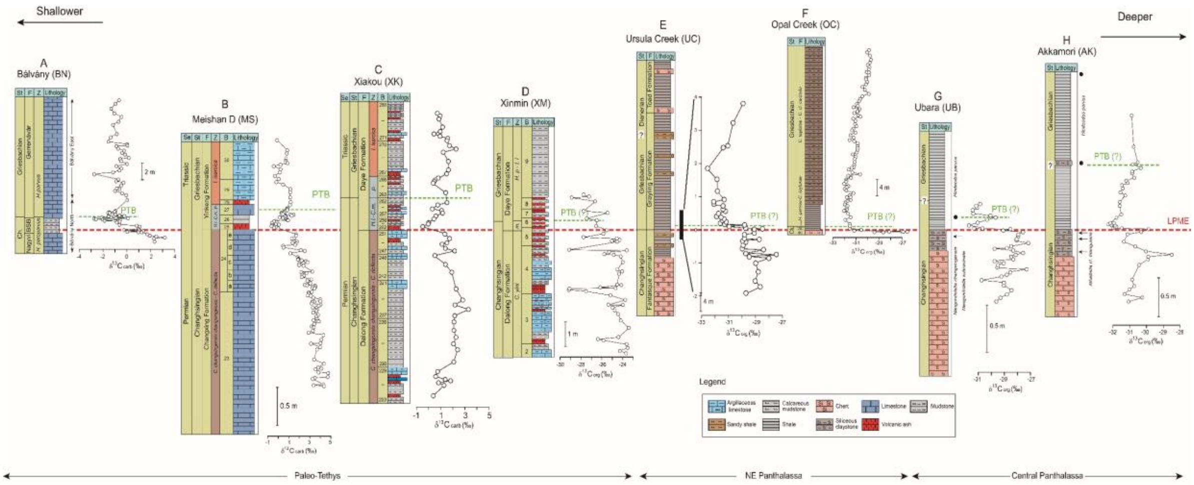


Supplementary Figure 14. Correlations among the sections. (A) Bálvány; (B) Meishan D; (C) Xiakou; (D) Xinmin; (E) Ursula Creek; (F) Opal Creek; (G) Ubara; (H) Akkamori 2. (Bio-)stratigraphic data of each section from: Bálvány[55]; Meishan D[32]; Xiakou[45]; Xinmin[45]; Ursula Creek[26]; Opal Creek[23]; Ubara[67]; Akkamori 2[74]. Carbon isotope data from: Bálvány[55]; Meishan D[40]; Xiakou[45]; Xinmin[45]; Ursula Creek[27]; Opal Creek[23]; Ubara[67]; Akkamori 2[74]. Other details refer to Figure 1.

`

**Supplementary References**


1. Schroeder WH, Munthe J. Atmospheric mercury—an overview. *Atmospheric Environment* **32**, 809-822 (1998).

2. Percival L*, et al.* Globally enhanced mercury deposition during the end-Pliensbachian extinction and Toarcian OAE: A link to the Karoo–Ferrar Large Igneous Province. *Earth and Planetary Science Letters* **428**, 267-280 (2015).

3. Selin NE. Global biogeochemical cycling of mercury: a review. *Annual Review of Environment and Resources* **34**, 43-63 (2009).

4. Mason RP, Fitzgerald WF, Morel FM. The biogeochemical cycling of elemental mercury: anthropogenic influences. *Geochimica et Cosmochimica Acta* **58**, 3191-3198 (1994).

5. Lamborg CH, Fitzgerald WF, O'Donnell J, Torgersen T. A non-steady-state compartmental model of global-scale mercury biogeochemistry with interhemispheric atmospheric gradients. *Geochimica et Cosmochimica Acta* **66**, 1105-1118 (2002).

6. Amyot M, Gill GA, Morel FM. Production and loss of dissolved gaseous mercury in coastal seawater. *Environmental Science & Technology* **31**, 3606-3611 (1997).

7. Lindberg SE*, et al.* Dynamic oxidation of gaseous mercury in the Arctic troposphere at polar sunrise. *Environmental Science & Technology* **36**, 1245-1256 (2002).

8. Clarkson TW. Mercury: major issues in environmental health. *Environmental Health Perspectives* **100**, 31-38 (1993).

9. Feng XB, Qiu GL. Mercury pollution in Guizhou, Southwestern China—an overview. *Science of the Total Environment* **400**, 227-237 (2008).

10. Sial AN*, et al.* Mercury as a proxy for volcanic activity during extreme environmental turnover: The Cretaceous–Paleogene transition. *Palaeogeography, Palaeoclimatology, Palaeoecology* **387**, 153-164 (2013).

11. Sial AN*, et al.* High-resolution Hg chemostratigraphy: A contribution to the distinction of chemical fingerprints of the Deccan volcanism and Cretaceous–Paleogene Boundary impact event. *Palaeogeography, Palaeoclimatology, Palaeoecology* **414**, 98-115 (2014).

12. Mason RP, Fitzgerald WF. The distribution and biogeochemical cycling of mercury in the equatorial Pacific Ocean. *Deep Sea Research Part I: Oceanographic Research Papers* **40**, 1897-1924 (1993).

13. Zhang Y, Jaeglé L, Thompson L. Natural biogeochemical cycle of mercury in

a global three-dimensional ocean tracer model. *Global Biogeochemical Cycles* **28**, 553-570 (2014).

14. Morel FM, Kraepiel AM, Amyot M. The chemical cycle and bioaccumulation of mercury. *Annual Review of Ecology and Systematics* **29**, 543-566 (1998).

15. Bergquist BA, Blum JD. Mass-dependent and-independent fractionation of Hg isotopes by photoreduction in aquatic systems. *Science* **318**, 417-420 (2007).

16. Blum JD, Sherman LS, Johnson MW. Mercury Isotopes in Earth and Environmental Sciences. *Annual Review of Earth and Planetary Sciences* **42**, 249-269 (2014).

17. Chen JB, Hintelmann H, Feng XB, Dimock B. Unusual fractionation of both odd and even mercury isotopes in precipitation from Peterborough, ON, Canada. *Geochimica et Cosmochimica Acta* **90**, 33-46 (2012).

18. Perrot V, Bridou R, Pedrero Z, Guyoneaud R, Monperrus M, Amouroux D. Identical Hg isotope mass dependent fractionation signature during methylation by sulfate-reducing bacteria in sulfate and sulfate-free environment. *Environmental Science & Technology* **49**, 1365-1373 (2015).

19. Henderson CM. Uppermost Permian conodonts and the Permian-Triassic boundary in the Western Canada sedimentary basin. *Bulletin of Canadian Petroleum Geology* **45**, 693-707 (1997).

20. Schoepfer SD, Henderson CM, Garrison GH, Ward PD. Cessation of a productive coastal upwelling system in the Panthalassic Ocean at the Permian–Triassic Boundary. *Palaeogeography, Palaeoclimatology, Palaeoecology* **313-314**, 181-188 (2012).

21. Algeo TJ, Twitchett RJ. Anomalous Early Triassic sediment fluxes due to elevated weathering rates and their biological consequences. *Geology* **38**, 1023-1026 (2010).

22. Algeo TJ, Henderson CM, Tong JN, Feng QL, Yin HF, Tyson RV. Plankton and productivity during the Permian–Triassic boundary crisis: An analysis of organic carbon fluxes. *Global and Planetary Change* **105**, 52-67 (2013).

23. Schoepfer SD*, et al.* Termination of a continent-margin upwelling system at the Permian–Triassic boundary (Opal Creek, Alberta, Canada). *Global and Planetary Change* **105**, 21-35 (2013).

24. Shen J*, et al.* Marine productivity changes during the end-Permian crisis and Early Triassic recovery. *Earth-Science Reviews* **149**, 136-162 (2015).

25. Farley K, Ward P, Garrison G, Mukhopadhyay S. Absence of extraterrestrial 3He in Permian–Triassic age sedimentary rocks. *Earth and Planetary Science Letters* **240**, 265-275 (2005).

26. Wignall PB, Newton R. Contrasting deep-water records from the Upper

Permian and Lower Triassic of South Tibet and British Columbia: evidence for a diachronous mass extinction. *Palaios* **18**, 153-167 (2003).

27. Wang K, Geldsetzer H, Krouse H. Permian-Triassic extinction: Organic δ13C evidence from British Columbia, Canada. *Geology* **22**, 580-584 (1994).

28. Wignall PB, Twitchett RJ. Extent, duration, and nature of the Permian-Triassic superanoxic event. *Geological Society of America Special Papers* **356**, 395-414 (2002).

29. Yang ZY, Yin HF, Wu SB, Yang FQ, Ding MH, Xu GR. Permian–Triassic boundary stratigraphy and fauna of South China. *Geological Publishing House, Beijing,* 378 p. (1987) (in Chinese with English abstract).

30. Feng QL, He WH, Gu SZ, Meng YY, Jin YX, Zhang F. Radiolarian evolution during the latest Permian in South China. *Global and Planetary Change* **55**, 177-192 (2007).

31. Yin HF, Jiang HS, Xia WC, Feng QL, Zhang N, Shen J. The end-Permian regression in South China and its implication on mass extinction. *Earth-Science Reviews* **137**, 19-33 (2014).

32. Yin HF, Zhang, KX, Tong, JN, Yang ZY, Wu SB. The global stratotype section and point (GSSP) of the Permian-Triassic boundary. *Episodes* **24**, 102-114 (2001).

33. Jin YG, Wang Y, Wang W, Shang QH, Cao CQ, Erwin DH. Pattern of marine mass extinction near the Permian-Triassic boundary in South China. *Science* **289**, 432-436 (2000).

34. Jiang HS, Lai XL, Luo GM, Aldridge R, Zhang KX, Wignall P. Restudy of conodont zonation and evolution across the P/T boundary at Meishan section, Changxing, Zhejiang, China. *Global and Planetary Change* **55**, 39-55 (2007).

35. Shen SZ*, et al.* Calibrating the end-Permian mass extinction. *Science* **334**, 1367-1372 (2011).

36. Burgess SD, Bowring SA. High-precision geochronology confirms voluminous magmatism before, during, and after Earth's most severe extinction. *Science Advances* **1**, e1500470 (2015).

37. Joachimski MM*, et al.* Climate warming in the latest Permian and the Permian-Triassic mass extinction. *Geology* **40**, 195-198 (2012).

38. Grice K*, et al.* Photic zone euxinia during the Permian-triassic superanoxic event. *Science* **307**, 706-709 (2005).

39. Xie SC, Pancost RD, Yin HF, Wang HM, Evershed RP. Two episodes of microbial change coupled with Permo/Triassic faunal mass extinction. *Nature* **434**, 494-497 (2005).

40. Xie SC*, et al.* Changes in the global carbon cycle occurred as two episodes during the Permian–Triassic crisis. *Geology* **35**, 1083-1086 (2007).

41. Cao CQ, Love GD, Hays LE, Wang W, Shen SZ, Summons RE. Biogeochemical evidence for euxinic oceans and ecological disturbance presaging the end-Permian mass extinction event. *Earth and Planetary Science Letters* **281**, 188-201 (2009).

42. Xie SC*, et al.* Cyanobacterial blooms tied to volcanism during the 5 m.y. Permo-Triassic biotic crisis. *Geology* **38**, 447-450 (2010).

43. Yin HF, Xie SC, Luo GM, Algeo TJ, Zhang KX. Two episodes of environmental change at the Permian–Triassic boundary of the GSSP section Meishan. *Earth-Science Reviews* **115**, 163-172 (2012).

44. Zhao LS, Chen YL, Chen ZQ, Cao L. Uppermost Permian to Lower Triassic Conodont Zonation from Three Gorges Area, South China. *Palaios* **28**, 523-540 (2013).

45. Shen J*, et al.* Negative C-isotope excursions at the Permian-Triassic boundary linked to volcanism. *Geology* **40**, 963-966 (2012).

46. Shen J, Algeo TJ, Hu Q, Xu GZ, Zhou L, Feng QL. Volcanism in South China during the Late Permian and its relationship to marine ecosystem and environmental changes. *Global and Planetary Change* **105**, 121-134 (2013).

47. Shen J*, et al.* Two pulses of oceanic environmental disturbance during the Permian–Triassic boundary crisis. *Earth and Planetary Science Letters* **443**, 139-152 (2016).

48. Wang GQ, Xia WC. Conodont zonation across the Permian-Triassic boundary at the Xiakou section, Yichang city, Hubei Province and its correlation with the Global Stratotype Section and Point of the PTB. *Canadian Journal of Earth Sciences* **41**, 323-330 (2004).

49. Shen J, Algeo T, Zhou L, Feng QL, Yu JX, Ellwood B. Volcanic perturbations of the marine environment in South China preceding the latest Permian mass extinction and their biotic effects. *Geobiology* **10**, 82-103 (2012).

50. Wei HY, Shen J, Schoepfer SD, Krystyn L, Richoz S, Algeo TJ. Environmental controls on marine ecosystem recovery following mass extinctions, with an example from the Early Triassic. *Earth-Science Reviews* **149**, 108-135 (2015).

51. Elrick M*, et al.* Global-ocean redox variation during the middle-late Permian through Early Triassic based on uranium isotope and Th/U trends of marine carbonates. *Geology* **45**, 163-166 (2017).

52. Shen J*, et al.* Volcanic Effects on Microplankton during the Permian-Triassic Transition (Shangsi and Xinmin, South China). *Palaios* **28**, 552-567 (2013).

53. Haas J, Hips K, Pelikán P, Zajzon N, Götz AE, Tardi-Filácz E. Facies analysis of marine Permian/Triassic boundary sections in Hungary. *Acta Geologica Hungarica* **47**, 297-340 (2004).

54. Haas J*, et al.* Biotic and environmental changes in the Permian–Triassic boundary interval recorded on a western Tethyan ramp in the Bükk Mountains, Hungary. *Global and Planetary Change* **55**, 136-154 (2007).

55. Schobben M*, et al.* Volatile earliest Triassic sulfur cycle: A consequence of persistent low seawater sulfate concentrations and a high sulfur cycle turnover rate? *Palaeogeography, Palaeoclimatology, Palaeoecology* **486**, 74-85 (2017).

56. Sudar M, Perri MC, Haas J. Conodonts across the Permian–Triassic boundary in the Bükk Mountains (NE Hungary). *Geologica Carpathica* **59**, 491-502 (2008).

57. Isozaki Y. Permo-Triassic Boundary Superanoxia and Stratified Superocean: Records from Lost Deep Sea. *Science* **276**, 235-238 (1997).

58. Maruyama S, Isozaki Y, Kimura G, Terabayashi M. Paleogeographic maps of the Japanese Islands: plate tectonic synthesis from 750 Ma to the present. *Island Arc* **6**, 121-142 (1997).

59. Algeo TJ*, et al.* Changes in productivity and redox conditions in the Panthalassic Ocean during the latest Permian. *Geology* **38**, 187-190 (2010).

60. Algeo TJ, Chen ZQ, Fraiser ML, Twitchett RJ. Terrestrial–marine teleconnections in the collapse and rebuilding of Early Triassic marine ecosystems. *Palaeogeography, Palaeoclimatology, Palaeoecology* **308**, 1-11 (2011).

61. Yao J, Yao A, Kuwahara K. Upper Permian biostratigraphic correlation between conodont and radiolarian zones in the Tamba-Mino Terranesouthwest Japan. *Journal of Geosciences Osaka City University* **44**, 97-119 (2001).

62. Xia WC, Zhang N, Wang GQ, Youshitaka K. Pelagic radiolarian and conodont biozonation in the Permo-Triassic boundary interval and correlation to the Meishan GSSP. *Micropaleontology* **50**, 27-44 (2004).

63. Algeo TJ*, et al.* Spatial variation in sediment fluxes, redox conditions, and productivity in the Permian–Triassic Panthalassic Ocean. *Palaeogeography, Palaeoclimatology, Palaeoecology* **308**, 65-83 (2011).

64. Takahashi S*, et al.* Bioessential element-depleted ocean following the euxinic maximum of the end-Permian mass extinction. *Earth and Planetary Science Letters* **393**, 94-104 (2014).

65. Takahashi S, Yamakita S, Suzuki N, Kaiho K, Ehiro M. High organic carbon content and a decrease in radiolarians at the end of the Permian in a newly discovered continuous pelagic section: A coincidence? *Palaeogeography,*

*Palaeoclimatology, Palaeoecology* **271**, 1-12 (2009).

66. Kimura K, Makimoto H, Yoshioka T. Geology of the Ayabe district. *Geological Survey of Japan* 104p. (1989).

67. Kaiho K*, et al.* Changes in depth-transect redox conditions spanning the end-Permian mass extinction and their impact on the marine extinction: Evidence from biomarkers and sulfur isotopes. *Global and Planetary Change* **94-95**, 20-32 (2012).

68. Korte C, Kozur HW. Carbon-isotope stratigraphy across the Permian–Triassic boundary: A review. *Journal of Asian Earth Sciences* **39**, 215-235 (2010).

69. Burgess SD, Bowring S, Shen SZ. High-precision timeline for Earth's most severe extinction. *Proceedings of the National Academy of Sciences of the United States of America* **111**, 3316-3321 (2014).

70. Sanei H, Grasby SE, Beauchamp B. Latest Permian mercury anomalies. *Geology* **40**, 63-66 (2012).

71. Grasby SE*, et al.* Isotopic signatures of mercury contamination in latest Permian oceans. *Geology* **45**, 55-58 (2017).

72. Grasby SE, Sanei H, Beauchamp B, Chen ZQ. Mercury deposition through the Permo–Triassic biotic crisis. *Chemical Geology* **351**, 209-216 (2013).

73. Grasby SE, Beauchamp B, Bond DP, Wignall PB, Sanei H. Mercury anomalies associated with three extinction events (Capitanian crisis, latest Permian extinction and the Smithian/Spathian extinction) in NW Pangea. *Geological magazine* **153**, 285-297 (2016).

74. Takahashi S, Kaiho K, Oba M, Kakegawa T. A smooth negative shift of organic carbon isotope ratios at an end-Permian mass extinction horizon in central pelagic Panthalassa. *Palaeogeography, Palaeoclimatology, Palaeoecology* **292**, 532-539 (2010).
